# Toward High‐Performance Hydrogenation at Room Temperature Through Tailoring Nickel Catalysts Stable in Aqueous Solution

**DOI:** 10.1002/advs.202309303

**Published:** 2024-04-06

**Authors:** Zidan Zou, Yue Shen, Xiao Zhang, Wenchao Li, Chun Chen, Diancai Fan, Haimin Zhang, Huijun Zhao, Guozhong Wang

**Affiliations:** ^1^ Key Laboratory of Materials Physics, Centre for Environmental and Energy Nanomaterials Institute of Solid State Phycis, HFIPS, Chinese Academy of Sciences 350 Shushanhu road Hefei 230031 China; ^2^ Science Island Branch Graduate School of USTC Hefei 230026 China; ^3^ Anhui Haoyuan Chemical Group Co., Ltd. Fuyang 236056 China; ^4^ Centre for Clean Environment and Energy Gold Coast Campus Griffith University Queensland 4222 Australia

**Keywords:** aqueous‐phase hydrogenation, multifunctional effects, nickel catalyst, nitrogen‐doped carbon–silica composite, room temperature

## Abstract

The development of highly active, reusable catalysts for aqueous‐phase reactions is challenging. Herein, metallic nickel is encapsulated in a nitrogen‐doped carbon–silica composite (SiO_2_@Ni@NC) as a catalyst for the selective hydrogenation of vanillin in aqueous media. The constructed catalyst achieved 99.8% vanillin conversion and 100% 4‐hydroxymethyl‐2‐methoxyphenol selectivity at room temperature. Based on combined scanning transmission electron microscopy, X‐ray photoelectron spectroscopy, and Raman analyses, the satisfactory catalytic performance is attributed to the composite structure consisting of an active metal, carbon, and silica. The hydrophilic silica core promoted dispersion of the catalyst in aqueous media. Moreover, the external hydrophobic NC layer has multiple functions, including preventing oxidation or leaching of the internal metal, acting as a reducing agent to reduce the internal metal, regulating the active‐site microenvironment by enriching the concentrations of H_2_ and organic reactants, and modifying the electronic structure of the active metal via metal–support interactions. Density functional theory calculations indicated that NC facilitates vanillin adsorption and hydrogen dissociation to promote aqueous‐phase hydrogenation. This study provides an efficient strategy for constructing encapsulated Ni‐based amphiphilic catalysts to upgrade biomass‐derived compounds.

## Graphical abstract

Metallic nickel nanoparticles encapsulated in nitrogen‐doped carbon‐silica composite amphiphilic support (SiO_2_@Ni@NC) can realize the complete hydrogenation of vanillin to 4‐hydroxymethyl‐2‐methoxyphenol in aqueous‐phase at room temperature. The hydrophilic silica core promotes the dispersion of the catalyst in water, while the hydrophobic NC surface promotes the enrichment and spontaneous dissociation of hydrogen molecules, and regulates the electron density of Ni species.

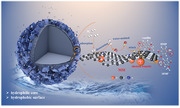

## 1 Introduction

Fossil fuel depletion and increasing greenhouse gas emissions are long‐standing problems. To overcome these issues, green and sustainable processes for producing energy and high‐value chemicals must be developed.^[^ ^1^, ^2^ ^]^ Biomass, a renewable energy source with abundant reserves on Earth, can be upgraded to high‐value chemicals and fuels using chemical and physical methods. As a cost‐effective and ecologically safe alternative to fossil fuels, biomass has potential for a wide range of applications with net‐zero carbon emissions.^[^ ^3^ ^]^ Although bio‐oils can be processed directly from raw biomass materials, the resulting bio‐oils often have high oxygen levels.^[^ ^4^ ^]^ Such oxygenates are of low value owing to their instability and low calorific values.^[^ ^5^, ^6^ ^]^ To obtain high‐value chemicals and fuels, bio‐oils must undergo further treatment, such as hydrogenation or hydrodeoxygenation (HDO), which are effective methods for reducing the oxygen content and promoting conversion into more stable alcohols and hydrocarbons.^[^ ^7^, ^8^, ^9^ ^]^

Vanillin, an important aromatic monomer derived from biomass, can be converted into 4‐hydroxymethyl‐2‐methoxyphenol (HMP) and 2‐methoxy‐4‐methylphenol (MMP) via hydrogenation and HDO.^[^ ^10^, ^11^ ^]^ Notably, HMP is an important compound for the production of food additives, perfumes, and pharmaceutical intermediates. Owing to their excellent activities, noble‐metal‐based catalysts are often used in hydrogenation reactions; however, their widespread application is hindered by the limited reserves of noble metals.^[^ ^12^ ^]^ In recent years, catalysts based on earth‐abundant non‐noble metals, including Ni, Cu, and Co, have received extensive attention.^[^ ^13^ ^]^ In particular, Ni‐based catalysts, which are inexpensive and have high activities, are promising for industrial applications.^[^ ^14^ ^]^ Moreover, simple strategies can be adopted to regulate the structure of Ni‐based catalysts, resulting in activities comparable to those of noble‐metal‐based catalysts.^[^ ^15^, ^16^ ^]^

As a green solvent for catalytic reactions, water is easy to access, inexpensive, nontoxic, and pollution free.^[^ ^17^ ^]^ Consequently, water utilization in catalytic reactions has garnered significant attention in recent years.^[^ ^18^, ^19^, ^20^ ^]^ For example, during CH_4_ oxidation, water molecules promote the direct conversion of CH_4_ to ^*^CH_3_OH by providing ^*^OH species, thus facilitating hydrogenation and the formation of CH_3_OH.^[^ ^21^ ^]^ Studies have also suggested that water as a solvent can form a unique water/metal interface (e.g., H_2_O/Au) to improve the catalytic performance during alcohol oxidation.^[^ ^22^ ^]^ Water molecules can promote the diffusion of protons on FeO (111) monolayer films in the absence of Lewis acid sites.^[^ ^23^ ^]^ In addition, water can promote hydrogen spillover on the surface of irreducible silica.^[^ ^24^ ^]^ In zeolite catalysts, water molecules regulate the microenvironment near the active sites and stabilize intermediates through hydrogen‐bond formation.^[^ ^25^ ^]^ Thus, water plays a pivotal role in promoting numerous catalytic processes by directly participating in reactions, stimulating hydrogen spillover, or forming hydrogen‐bonded structures.

However, catalytic reactions involving water are severely limited by difficulties in stabilizing the active metal species. Significant efforts have been made to maintain the activities of metal nanoparticles (NPs) without leaching or loss.^[^ ^4^, ^26^, ^27^, ^28^, ^29^ ^]^ Various studies have shown that metal encapsulation can effectively mitigate the loss of active components. For example, Hu et al. adopted a vacuum‐assisted impregnation strategy to prepare a hollow carbon‐sphere‐encapsulated nickel catalyst. This catalyst exhibited excellent performance for the aqueous‐phase hydrogenation–rearrangement tandem reaction and maintained its activity over ten cycles.^[^ ^18^ ^]^ Wang et al. reported a catalyst consisting of Pd clusters encapsulated in silicalite‐1 zeolite, which exhibited good stability against sintering at 600 °C under a H_2_ or O_2_ atmosphere and in the presence of water.^[^ ^30^ ^]^ In addition, confining metal NPs can effectively prevent further growth or aggregation and regulate the electronic properties.^[^ ^26^, ^31^ ^]^

Notably, the low solubility of hydrogen in water limits mass transfer during catalytic reactions.^[^ ^32^, ^33^ ^]^ Low hydrogen solubility can be circumvented by increasing the hydrogen pressure, but this approach poses safety hazards and wastes resources.^[^ ^34^ ^]^ Alternatively, the affinity with gas molecules can be enhanced by introducing hydrophobic functional groups through support modification or constructing a hydrophobic layer.^[^ ^35^, ^36^ ^]^ For example, Wakerley et al. designed a hydrophobic coating on dendritic Cu via treatment with 1‐octadecanethiol, and the submerged hydrophobic surfaces efficiently trapped numerous gas molecules at the nanoscale.^[^ ^37^ ^]^ For nanostructured materials, surface hydrophobicity treatments can lead to gas capture phenomena.^[^ ^38^ ^]^ Therefore, constructing a hydrophobic layer to encapsulate active metals is an effective approach for stabilizing catalysts and improving the surface hydrogen concentration, thereby facilitating catalytic reactions.

The preparation of amphiphilic catalysts with a hydrophilic core and hydrophobic surface is a promising strategy for achieving improved performance during aqueous‐phase catalysis. Gas molecules can pass through a carbon layer with a porous structure without any diffusion restrictions.^[^ ^18^, ^31^ ^]^ Moreover, the intrinsic hydrophobicity of carbon can modify the wettability and reactant enrichment capacity of a catalyst.^[^ ^39^ ^]^ Thus, a catalyst with a hydrophilic core and hydrophobic surface can be constructed by wrapping mesoporous carbon around a supported active metal. Notably, the mesoporous carbon layer can also serve as a reducing agent for metal oxides in close contact without requiring a H_2_ atmosphere. Amphiphilic catalysts, which have both hydrophilic and hydrophobic regions, can facilitate the formation of stable homogeneous dispersions in aqueous media.^[^ ^40^ ^]^ Furthermore, the interactions between water and other water molecules in the bulk aqueous phase are more thermodynamically favorable than wetting the hydrophobic surface of an amphiphilic catalyst. Consequently, the catalyst surface remains available for the adsorption of gas molecules.^[^ ^38^ ^]^ Unlike modification with organic functional groups, wrapping with a carbon layer results in metal–support interactions (MSIs) that change the electronic structure of the inner active metal. Specifically, the metal NPs provide electrons to electron‐deficient carbon, thereby promoting the adsorption of reactants.^[^ ^31^ ^]^

In this study, we encapsulated metallic nickel in a nitrogen‐doped carbon and silica composite amphiphilic support (SiO_2_@Ni@NC) as a catalyst for the selective hydrogenation of vanillin in the aqueous phase. The hydrophilic SiO_2_ core enabled dispersion of the catalyst in aqueous media, thereby facilitating contact with the reactants. As the hydrophobic surface, the NC layer had four major effects: 1) protecting the internal metal NPs from oxidation or leaching, 2) acting as a reducing agent to reduce the internal metal, 3) regulating the active‐site microenvironment by enriching the concentrations of H_2_ and organic reactants, and 4) acting as an electron modifier for the active metal. Owing to these synergistic effects, the optimized amphiphilic SiO_2_@Ni@NC catalyst achieved ≈100% conversion of vanillin to HMP during aqueous‐phase hydrogenation at room temperature. In addition, the designed catalyst demonstrated good stability over five cycles without activity loss and displayed universal applicability for the catalytic conversion of various unsaturated aldehydes via aqueous‐phase hydrogenation. Density functional theory (DFT) calculations were employed to investigate the role of the NC layer in facilitating reactant adsorption and hydrogen dissociation, and to elucidate the catalytic mechanism for the aqueous‐phase hydrogenation of vanillin. These findings provide a reference for the development of efficient and stable amphiphilic catalysts for upgrading biomass to high‐value chemicals and fuels.

## 2 Results and Discussion

### 2.1 Catalyst Characterization

**Figure 1:**
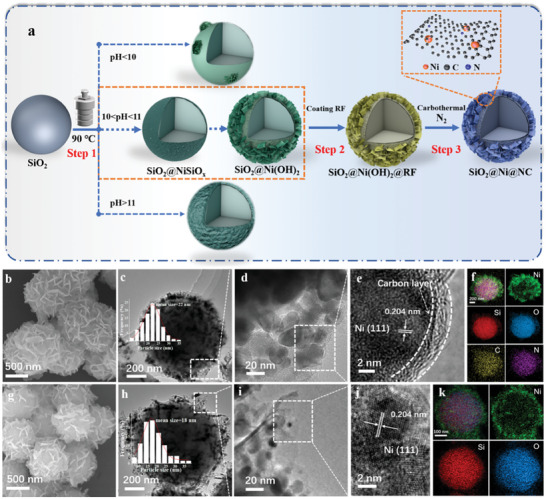
a) Schematic illustration of the synthesis procedure for SiO_2_@Ni@NC; b) SEM, c,d) TEM, e) HRTEM, and f) EDS mapping images of SiO_2_@Ni@NC; g) SEM, h,i) TEM, j) HRTEM, and k) EDS mapping images of SiO_2_@Ni.

Metallic Ni NPs were encapsulated in the ultrathin NC layer of a carbon–silica composite to achieve amphiphilic properties and significantly enhance the catalytic performance for aqueous‐phase hydrogenation. The synthesis of the amphiphilic catalyst can be divided into three steps (**Figure** **1**a). As a template, uniform silica colloidal particles with diameters of ≈700 nm were synthesized using the Stöber method. The surfaces of the silica spheres were relatively smooth without pores (Figure S1a, Supporting Information). Subsequently, nickel silicate was grown on the silica surface under ammonia etching and hydrothermal conditions. This rough nickel silicate layer acted as a growth and adhesion point for Ni(OH)_2_ nanosheets, which promoted the construction of a rough thin layer of Ni(OH)_2_ nanosheets on the silica colloidal particles. The SiO_2_@Ni(OH)_2_ precursor was then coated with a resorcinol–formaldehyde resin (RF) layer, which was formed by polycondensation (Figure 1a; Figure S1b, Supporting Information). Finally, the RF layer was carbonized under a high‐temperature inert atmosphere. The RF‐coated Ni(OH)_2_ nanosheets were reduced in situ to generate highly dispersed Ni NPs via a carbothermal reduction reaction, thereby forming the SiO_2_@Ni@NC catalyst. The thermogravimetric (TG) curve for the carbonization process is shown in Figure S2a (Supporting Information). The weight loss below 300 °C was mainly due to dehydration and the loss of small molecules from the RF layer, whereas the weight loss above 300 °C corresponded to RF carbonization. By regulating the RF coating process, the NC layer thickness could be adjusted in the range of 2–9 nm. SiO_2_@Ni@NC refers to the catalyst with a NC layer thickness of 3 nm.

Scanning electron microscopy (SEM) was used to examine the morphologies of the obtained samples. The dispersion of the Ni NPs was closely related to the structure of the Ni(OH)_2_ nanosheets, which differed significantly depending on the alkaline conditions during the hydrothermal step (Figure 1a; Figure S3, Supporting Information). Under weakly alkaline conditions (pH < 10.0), Ni(OH)_2_ nanosheets could not be easily generated, and only a small fraction of the Ni ions precipitated with ammonia to form Ni(OH)_2_. In contrast, strongly alkaline conditions (pH > 11.0) induced the formation of a dense nickel silicate layer, which hindered the subsequent deposition of a carbon coating. Consequently, a well‐constructed layer of Ni(OH)_2_ nanosheets was only obtained in the narrow pH range of 10.0–11.0. Thus, alkalinity played a critical role in the formation of well‐distributed Ni(OH)_2_ nanosheets. Notably, the particle size increased from 700 nm for SiO_2_ to 900 nm for SiO_2_@Ni(OH)_2_. This increase was due to the formation of the Ni(OH)_2_ nanosheet layer, suggesting that this layer had a thickness of ≈100 nm. To confirm this result, the SiO_2_ core in SiO_2_@Ni(OH)_2_ was selectively etched with alkali to form a hollow Ni(OH)_2_ shell structure, which after carbothermal treatment had a thickness of 100 nm (Figure S1c, Supporting Information). SiO_2_@Ni(OH)_2_@RF, obtained by coating RF on the surface of the Ni(OH)_2_ nanosheets, retained the morphology of SiO_2_@Ni(OH)_2_ (Figure S1b, Supporting Information).

When the SiO_2_@Ni(OH)_2_@RF precursor was transformed into SiO_2_@Ni@NC via carbothermal reduction, the morphology was well preserved (Figure 1b). TG analysis revealed that the carbon content in SiO_2_@Ni@NC was less than 2% (Figure S2b, Supporting Information), which is similar to the value obtained by organic elemental analysis (Table S1, Supporting Information, entry 3), indicating that the formed NC layer was ultrathin. The internal structures and metal distributions of the SiO_2_@Ni@NC and SiO_2_@Ni samples were characterized using transmission electron microscopy (TEM). In SiO_2_@Ni@NC, Ni NPs with an average size of ≈22 nm were evenly distributed between the amorphous SiO_2_ support and ultrathin NC layer (Figure 1c–e). High‐resolution transmission electron microscopy (HRTEM) revealed a crystal plane spacing of 0.204 nm for the Ni NPs, corresponding to a Ni (111) surface.^[^ ^41^ ^]^ Thus, the Ni species in the precursor were transformed into the metallic state through carbothermal treatment, which indirectly confirms the existence of a reducing carbon layer. A crystal plane spacing of 0.340 nm was also observed, which agrees well with the (002) crystal plane of carbon.^[^ ^42^ ^]^ The average thickness of the NC layer on the SiO_2_@Ni@NC catalyst was ≈3 nm. Elemental mapping analysis using energy‐dispersive X‐ray spectroscopy (EDS) revealed the distributions of Si, O, Ni, N, and C in the SiO_2_@Ni@NC catalyst (Figure 1f). Si was mainly distributed in the core of the hierarchically structured nanospheres, whereas Ni was evenly distributed on the exterior of the nanospheres. The distribution of N was similar to that of C, indicating that the N doping mainly originated from RF. Notably, the morphology of SiO_2_@Ni did not differ significantly from that of SiO_2_@Ni@NC (Figure 1g,h), although HRTEM observations confirmed the absence of graphitized NC in SiO_2_@Ni (Figure 1i,j). EDS also revealed homogeneous distributions of Si, O, and Ni in SiO_2_@Ni (Figure 1k) without any N or C.

**Figure 2:**
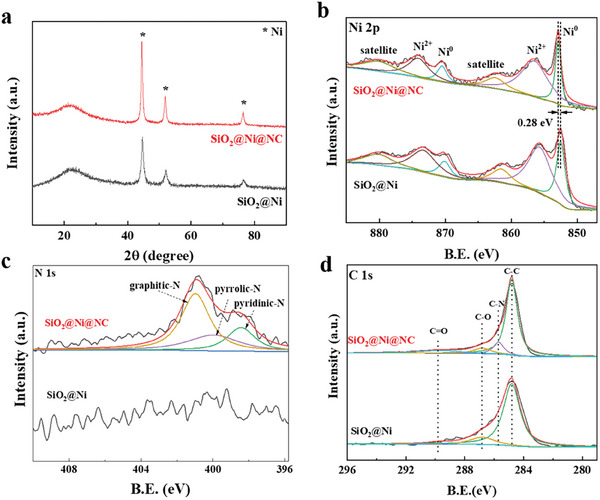
a) XRD patterns of SiO_2_@Ni@NC and SiO_2_@Ni; b) Ni 2p, c) N 1s, and d) C 1s XPS spectra of SiO_2_@Ni@NC and SiO_2_@Ni.

The X‐ray diffraction (XRD) patterns of the as‐prepared SiO_2_@Ni@NC and SiO_2_@Ni samples are shown in **Figure** **2**a. The diffraction peak at ≈22° was attributed to the characteristic peak of amorphous SiO_2_. The diffraction peaks at 44.5°, 51.8°, and 76.4° were indexed to the Ni (111), Ni (200), and Ni (220) planes, respectively, corresponding to the Ni (fcc) crystal phase. In comparison, the SiO_2_@Ni(OH)_2_@RF and SiO_2_@Ni(OH)_2_ precursors mainly exhibited diffraction peaks corresponding to Ni(OH)_2_ with some weak peaks attributed to NiSiO_x_ (Figure S2c, Supporting Information). N_2_ adsorption–desorption isotherms were used to investigate the specific surface areas and pore structures of the as‐prepared samples (Figure S4, Supporting Information). SiO_2_@Ni@NC and SiO_2_@Ni both exhibited type‐IV isotherms, indicating that the samples had highly mesoporous structures. Furthermore, SiO_2_@Ni@NC and SiO_2_@Ni had similar specific surface areas (101.9 and 102.4 m^2^ g^−1^, respectively), indicating that the NC layer on the surface of SiO_2_@Ni@NC was ultrathin and uniform with no detrimental effect on the pore structure. Raman spectroscopy also confirmed the presence of carbonaceous deposits in the SiO_2_@Ni@NC sample (Figure S5a, Supporting Information). The intensity ratio of the D and G peaks (*I*_D_/*I*_G_) of SiO_2_@Ni@NC was 2.57, indicating that the catalyst contained abundant defects.

The surface elements and chemical states of SiO_2_@Ni@NC and SiO_2_@Ni were evaluated using X‐ray photoelectron spectroscopy (XPS). As shown in Figure S5b (Supporting Information), Ni, O, and Si coexisted in both samples, consistent with the TEM results (Figure 1f,k). However, the intensities of the Ni, O, and Si peaks for SiO_2_@Ni@NC were weaker than those for SiO_2_@Ni because of the coating effect of the NC layer. In the Ni 2p spectrum of SiO_2_@Ni@NC (Figure 2b), two major peaks were observed at binding energies of 852.8 and 870.5 eV, corresponding to Ni 2p_3/2_ and Ni 2p_1/2_, respectively, which were ascribed to metallic Ni. The two peaks at 856.1 and 873.7 eV were attributed to the 2p_3/2_ and 2p_1/2_ states of Ni^2+^, respectively, and two satellite peaks were observed at 862.0 and 879.7 eV (Figure 2b). Interestingly, the Ni 2p_3/2_ peaks of SiO_2_@Ni@NC were shifted to higher binding energies relative to those of SiO_2_@Ni, indicating that the surface electron density of Ni was decreased by interactions between Ni and NC. Electron transfer from the metal to NC can increase the electron density of the NC layer, thereby promoting substrate adsorption and facilitating reactions between the substrate and active hydrogen dissociated from the active metal. In the C 1s spectrum (Figure 2d), the four peaks at 284.8, 285.7, 285.8, and 289.6 eV were assigned to C─C, C─N, C─O, and C═O groups, respectively, suggesting the successful coating of a carbon layer on the surface of SiO_2_@Ni@NC. Furthermore, in the N 1s spectrum (Figure 2c), the peaks at 398.6, 400.1, and 401.1 eV were assigned to pyridinic‐N, pyrrolic‐N, and graphitic‐N, respectively. Thus, the combined C and N spectra indicated that N was successfully doped into the carbon matrix to form a NC layer. Conversely, the C 1s and N 1s spectra of SiO_2_@Ni revealed the absence of C and N.

### 2.2 Catalytic Performance for Vanillin Hydrogenation

**Figure 3:**
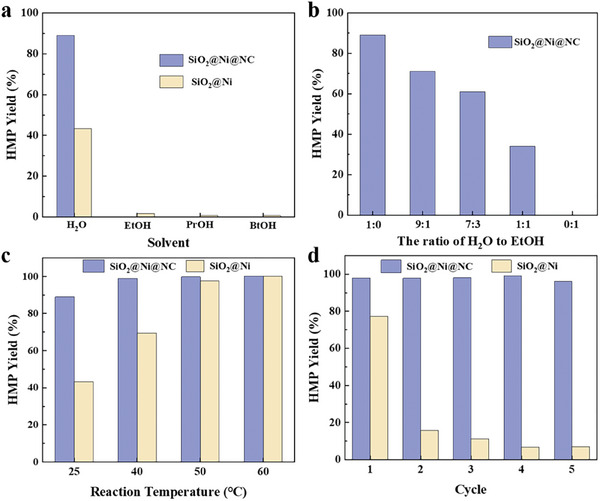
a) HMP yields for vanillin hydrogenation in different solvents (25 °C, 2.0 MPa H_2_, 2 h, 1 mmol of vanillin, 10 mL of solvent, and 30 mg of catalyst); b) effect of water and ethanol volume ratios on vanillin hydrogenation over SiO_2_@Ni@NC (25 °C, 2.0 MPa H_2_, 2 h, 1 mmol vanillin, 10 mL solvent, and 30 mg catalyst); c) HMP yields for vanillin hydrogenation at different reaction temperatures over SiO_2_@Ni@NC and SiO_2_@Ni (2.0 MPa H_2_, 2 h, 1 mmol of vanillin, 10 mL of water, and 30 mg of catalyst); d) Recycling performance for vanillin conversion over SiO_2_@Ni@NC and SiO_2_@Ni.

In lignocellulosic biomass, lignin is the only renewable energy source that can be used to produce aromatic compounds. As an important aromatic monomer derived from lignin, vanillin can be further transformed into high‐value aromatic compounds or intermediates via hydrogenation reactions.^[^ ^10^, ^11^ ^]^ In this study, vanillin was chosen as a model reactant to explore the catalytic hydrogenation performance of as‐synthesized SiO_2_@Ni@NC under various reaction conditions (**Figure** **3**; Figure S6 and Table S2, Supporting Information).

The catalytic performance of the SiO_2_@Ni@NC catalyst for the aqueous‐phase hydrogenation of vanillin was evaluated at room temperature and compared with that of the sample without carbon encapsulation (SiO_2_@Ni). As shown in Figure 3a and Table S3 (Supporting Information), the SiO_2_@Ni@NC catalyst achieved an HMP yield of 89.2% and turnover frequency (TOF) of 20.7 h^−1^, which is more than twice that of SiO_2_@Ni. In addition, the SiO_2_@Ni(OH)_2_ and SiO_2_@Ni(OH)_2_@RF precursors showed no catalytic activity (Table S2, Supporting Information, entries 1 and 2), indicating that the Ni species must be transformed into metallic Ni to obtain active sites for aqueous‐phase hydrogenation. According to the TEM, XRD, and CO chemisorption results (Figures 1c,h and 2a; Figure S7 and Table S3, Supporting Information), the size and dispersion of metallic Ni were similar in the SiO_2_@Ni@NC and SiO_2_@Ni catalysts. Thus, these properties are not the main factors responsible for the improved activity of SiO_2_@Ni@NC. A comparison of the structures of SiO_2_@Ni@NC and SiO_2_@Ni suggests that the enhanced catalytic performance is mainly due to the coating effect of the NC layer.

Notably, the catalytic activities of both SiO_2_@Ni@NC and SiO_2_@Ni were higher in the aqueous phase than in the organic phase. At room temperature in the aqueous‐phase reaction system, vanillin was effectively hydrogenated to form HMP. In contrast, the organic‐phase reaction system showed little activity, indicating that water plays an important role in vanillin hydrogenation under mild reaction conditions. Water has been widely reported to play beneficial roles in catalytic reactions, including forming hydrogen‐bond networks to facilitate hydrogen spillover, adjusting the active‐site microenvironment, and building unique water/metal interfaces.^[^ ^22^, ^23^ ^]^ To explore the role of water in vanillin hydrogenation over SiO_2_@Ni@NC, various mixtures water and ethanol (a protonic solvent) were used as the reaction media (Figure 3b). The optimal activity was observed in the pure water system. The catalytic activity decreased with increasing ethanol content, with minimal activity observed in the pure organic solvent system. In protonic solvents, organic reactants have good miscibility with the solvent, resulting in a solvation effect. Each reactant molecule is tightly surrounded by a solvation shell with one or several layers. During heterogeneous hydrogenation, the catalyst must penetrate the solvation shell to encounter the reactant molecules. Competitive adsorption occurs between the solvent and reactant molecules on the partially graphitized NC layer. The cage effect of the solvent and competitive adsorption results in poor catalytic performance in protonic solvents. In contrast, organic reactant molecules are weakly hydrated in aqueous‐phase catalysis, allowing the catalyst to easily penetrate the solvation layer, which results in enhanced adsorption and activation of the reactant molecules. In addition, the hydrophilic SiO_2_ core provides the catalyst with overall hydrophilicity, allowing uniform dispersion in the water system. Similar to the solvation effect, this phenomenon can significantly increase the probability of collisions between the catalyst and reactant molecules. Moreover, the partially graphitized NC layer has a strong adsorption capacity for organic reactant molecules, but weakly adsorbs water molecules, thereby reducing the influence of competitive adsorption. These factors promote the catalytic performance in the aqueous phase, which is superior to that in organic systems. This effect was further verified by the reaction behavior in the mixed water and ethanol systems. The introduction of water can break up the solvent cage to facilitate contact between the catalyst and reactant molecules. Thus, the reaction activity gradually increases with increasing water content.

Critically, protonic solvents can form hydrogen‐bond networks, which facilitate the transfer of active hydrogen to the active sites of the catalyst during hydrogenation.^[^ ^23^, ^43^ ^]^ The hydrogen‐bond network consists of hydrogen bonds with the form of [R─O(H)^…^H─O(R)^…^H─O(R)], where R is H in water and an organic group in protonic alcohols. In the hydrogen‐bond network of water, the surface charge of hydrogen can be separated to act as a proton, which can hop to an adjacent water molecule to form a hydronium ion, causing a proton in that water molecule to be transferred to another neighboring water molecule. This well‐known Grotthuss mechanism is conducive to reducing the activation barrier for carbonyl hydrogenation.^[^ ^44^, ^45^ ^]^ To explore whether stronger hydrogen bonding results in better hydrogenation performance, we employed heavy water as the solvent for vanillin hydrogenation (Figure S8, Supporting Information). The activity in heavy water was greater than that in organic solvents but weaker than that in water. Interestingly, the catalytic activity was inversely proportional to the hydrogen‐bond strength in water and heavy water (22 and 29 kJ mol^−1^, respectively). The distribution of hydrogenated produced observed in the ^1^H nuclear magnetic resonance (NMR) spectra were consistent with these results (Figure S9, Supporting Information), indicating that an appropriate hydrogen bond strength is beneficial for catalytic reactions in the liquid phase. Therefore, hydrogen bonds of moderate strength and a uniform network structure are crucial for promoting the hydrogenation reaction. Thus, we inferred that the hydrogen‐bond network in the aqueous phase promoted vanillin hydrogenation on the active sites of the SiO_2_@Ni@NC and SiO_2_@Ni catalysts.

In addition to the solvent, temperature has a crucial effect on the vanillin hydrogenation activity. The effect of the reaction temperature on vanillin hydrogenation was investigated at room temperature to 120 °C with a H_2_ pressure of 2 MPa (Figure 3c; Figure S10, Supporting Information). The SiO_2_@Ni@NC catalyst showed high activity at room temperature with a relatively low hydrogen pressure, indicating excellent hydrogenation performance. The conversion of vanillin gradually increased as the reaction temperature was increased to 50 and 60 °C. Overall, a desirable conversion of ≈100% with high HMP selectivity was achieved at low reaction temperatures of 25–60 °C (Figure 3c). Further increasing the temperature to 80 °C greatly improved the catalytic activity, and the generated carbon–oxygen single bonds could be further activated and broken, resulting in HDO reactions (Figure S10, Supporting Information). Under these conditions, the carbonyl group in the branched chain of vanillin is activated and hydrogenated to form a hydroxyl group, thereby generating HMP. In a subsequent HDO reaction, the hydroxyl group is further transformed into a methyl group to generate MMP. The MMP yield gradually increased and the HMP yield decreased with increasing reaction temperature. In the entire reaction temperature range, the catalytic performance of SiO_2_@Ni@NC was superior to that of SiO_2_@Ni, suggesting that the NC layer has a significant enhancement effect, which is consistent with the activation energy results (Figure S11, Supporting Information).

To verify the importance of the NC layer in the hydrogenation reaction, we performed UV–vis and temperature‐programmed desorption of H_2_ (H_2_‐TPD) tests. As shown in Figure S12 (Supporting Information), the SiO_2_@Ni@NC catalyst had a higher adsorption capacity for vanillin than the SiO_2_@Ni catalyst, which was attributed to the enrichment of vanillin molecules by the NC layer. In addition, the NC layer may help capture hydrogen molecules in the reaction system. As shown in the H_2_‐TPD profiles (Figure S6c, Supporting Information), both samples exhibited several desorption peaks in the temperature ranges of 50–150 and 300–550 °C. The low‐temperature range (50–150 °C) represented the desorption of weakly chemisorbed H_2_ molecules on the exposed Ni sites. In contrast, the high‐temperature region (300–550 °C) was assigned to strongly chemisorbed H_2_ molecules, such as those adsorbed at the metal/support interface. For SiO_2_@Ni@NC, a notable desorption peak appeared at ≈473 °C, indicating that the H_2_ adsorption strength on SiO_2_@Ni@NC is greater than that on SiO_2_@Ni. The difficulty in desorbing H_2_ is likely due to H_2_ being adsorbed at the interface between the Ni sites and NC layer. In addition, the total H_2_ adsorption peak area was larger for SiO_2_@Ni@NC than for SiO_2_@Ni, suggesting that the hydrogen enrichment capacity of SiO_2_@Ni@NC is greater than that of SiO_2_@Ni, reflecting the stronger affinity between SiO_2_@Ni@NC and H_2_. Based on these results, the NC layer not only significantly enriched organic species but could also increase the hydrogen concentration.

In addition to regulating activity and selectivity, the NC layer significantly enhanced the stability of the Ni catalyst during aqueous‐phase hydrogenation. After five cycles, the activity of the SiO_2_@Ni@NC catalyst was maintained, whereas that of the SiO_2_@Ni catalyst was reduced to one‐tenth of its initial activity. The excellent stability of the SiO_2_@Ni@NC catalyst was mainly attributed to the protective effect of the NC layer. No obvious changes were observed in the SEM images, TEM images, and XRD patterns of SiO_2_@Ni@NC before and after use (Figures S13 and S14a, Supporting Information), and the inductively coupled plasma‐atomic emission spectroscopy (ICP‐AES) results confirmed that only trace Ni was lost after five cycles (Table S4, Supporting Information, entry 3). These observations suggest that the presence of the hydrophobic NC layer effectively inhibits the leaching of Ni NPs during the aqueous‐phase reaction. Based on the XRD and ICP‐AES results, the poor performance of the SiO_2_@Ni catalyst was caused by Ni leaching (Figure S14b and Table S4, Supporting Information, entry 4). Therefore, the NC coating on the surface of the catalyst can regulate the activity, selectivity, and stability of the Ni catalyst. Thus, SiO_2_@Ni@NC achieved good catalytic performance for the aqueous‐phase hydrogenation of vanillin. Notably, this catalyst also exhibited outstanding performance in a gram‐scale experiment (Figure S15, Supporting Information).

### 2.3 Effect of NC on Catalytic Activity

As mentioned above, the NC layer endows the Ni catalyst with room‐temperature activity, good selectivity, and excellent stability. Here, we performed an in‐depth investigation of the positive effect of NC on aqueous‐phase hydrogenation.

**Figure 4:**
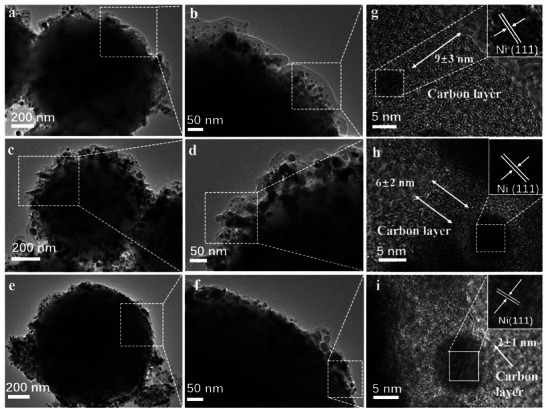
TEM images of a,b) SiO_2_@Ni@NC‐9, c,d) SiO_2_@Ni@NC‐6, and e,f) SiO_2_@Ni@NC‐2; HRTEM images of g) SiO_2_@Ni@NC‐9, h) SiO_2_@Ni@NC‐6, and i) SiO_2_@Ni@NC‐2.

Based on the formation mechanism shown in Figure 1a, the thickness of the NC layer is tunable. NC layer thicknesses of 2, 3, 6, and 9 nm were obtained by adjusting the RF coating amount. SiO_2_@Ni@NC represents the catalyst with a NC layer thickness of 3 nm. The catalysts with NC layer thicknesses of 2, 6, and 9 nm were denoted as SiO_2_@Ni@NC‐*x*, where *x* = 2, 6, and 9. The change in NC layer thickness can be directly observed in the scanning transmission electron microscopy (STEM) images (Figures 1e and **4**g–i). The Ni NPs were dispersed uniformly on the nanosheets in all of the SiO_2_@Ni@NC‐*x* catalysts (Figure 4a–f; Table S4, Supporting Information). As shown in Figure S16 (Supporting Information), SiO_2_@Ni@NC‐2, SiO_2_@Ni@NC‐6, and SiO_2_@Ni@NC‐9 had specific surface areas of 101.9, 116.4, and 121.5 m^2^ g^−1^, respectively, indicating that increasing the thickness of the NC layer slightly increases the specific surface area. As evidenced by the XRD patterns (Figure S17a, Supporting Information), the diffraction intensities of Ni were similar for all the samples.

**Figure 5:**
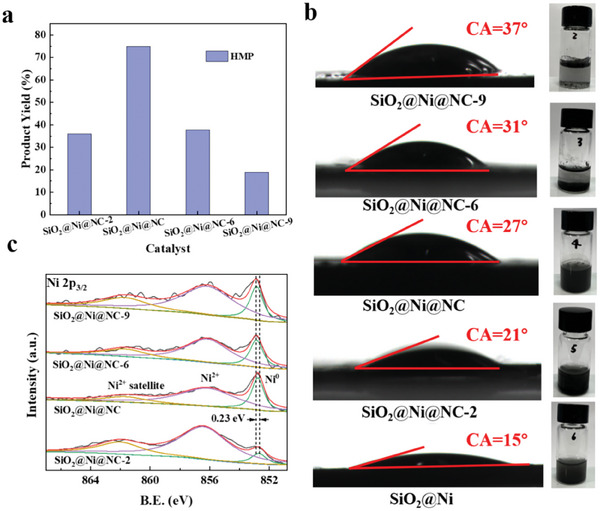
a) HMP yields for vanillin hydrogenation over SiO_2_@Ni@NC catalysts with different NC layer thicknesses (25 °C, 3.5 MPa H_2_, 1 h, 1 mmol of vanillin, 10 mL of water, and 30 mg of catalyst); b) water contact angles of SiO_2_@Ni@NC catalysts with different NC layer thicknesses; c) Ni 2p XPS spectra of SiO_2_@Ni@NC catalysts with different NC layer thicknesses.

We applied the SiO_2_@Ni@NC‐*x* catalysts to the aqueous‐phase hydrogenation of vanillin to explore the effect of the NC layer thickness (**Figure** **5**a). The activity of SiO_2_@Ni@NC was superior to those of SiO_2_@Ni@NC‐2, SiO_2_@Ni@NC‐6, and SiO_2_@Ni@NC‐9. The HMP yield obtained with SiO_2_@Ni@NC was more than twice that observed with the other samples.

H_2_‐TPD was used to investigate the effect of the NC layer on H_2_ absorption by the active species (Figure S17b, Supporting Information). The H_2_ desorption profiles comprised three temperature regions: 50–150, 300–450, and 450–550 °C. All the samples exhibited H_2_ desorption peaks in both the 50–150 and 300–450 °C regions, which corresponded to weakly adsorbed and strongly chemisorbed H_2_ on the Ni sites, respectively. In the high‐temperature region, the peak intensity generally increased with increasing NC layer thickness, indicating that more H_2_ was adsorbed on thicker NC layers. The interactions between the catalyst and H_2_ were improved by the superaerophilic properties of the thicker NC layers, which facilitate H_2_ adsorption, resulting in H_2_ enrichment in the environment surrounding the active sites.^[^ ^34^ ^]^ Considering the low solubility of H_2_ in water, H_2_ enrichment by the NC layer can ensure a supply of active hydrogen species near the active sites during aqueous‐phase hydrogenation, which is expected to enhance the catalytic activity.

The wettability of the catalyst is crucial for catalytic reactions in water. To determine the effect of the hydrophobic NC layer on catalyst wettability, contact angle experiments were conducted. As shown in Figure 5b, the sample without a NC layer exhibited superhydrophilicity, with a water contact angle of 15°. The hydrophilicity gradually decreased with increasing NC layer thickness. The change in the water contact angle induced by the catalyst wettability led to differences in catalytic performance. Interestingly, because SiO_2_@Ni@NC was amphiphilic with a hydrophilic core and hydrophobic surface, this catalyst retained hydrophilic properties. The hydrophilicity of the amphiphilic SiO_2_@Ni@NC catalyst promoted dispersion and ensured access to dissolved reactants in the aqueous‐phase system. In contrast, the hydrophobic NC layer facilitated the adsorption of organic compounds, resulting in reactant enrichment on the active sites of the catalyst.^[^ ^46^ ^]^ Raman spectroscopy revealed that SiO_2_@Ni@NC had a higher *I*_D_/*I*_G_ value (2.57) than the other samples (Figure S17c, Supporting Information), suggesting the presence of a large number of defect sites, which can adsorb reaction substrates and thus promote catalytic performance. In this case, upon releasing the dissociated active hydrogen species, the reactant undergoes immediate hydrogenation, which greatly enhances the reaction efficiency. Therefore, the thickness of the NC layer is a key factor in the catalytic performance during aqueous‐phase hydrogenation.

An excessively thin NC layer did not effectively reduce the inner Ni species to metallic Ni. As shown in Figure 5c, SiO_2_@Ni@NC‐2 had the lowest metallic Ni content. As the thickness of the NC layer increases, the barrier for reactant and H_2_ transport across the NC layer increases, and mass transfer becomes the rate‐limiting step of the catalytic reaction. Therefore, constructing a suitable NC layer is an effective strategy to avoid mass‐transfer limitations during the catalytic reaction and accelerate hydrogenation.^[^ ^18^ ^]^ In addition, the NC layer modifies the electronic properties of the reduced Ni species, which also influences the hydrogenation performance. Increasing the thickness of the NC layer caused Ni 2p peak corresponding to metallic Ni to shift toward a higher binding energy (Figure 5c). This behavior suggests that electron transfer occurs between the NC layer and Ni species, resulting in electron‐deficient Ni and electron‐rich NC. As the metal is reduced in situ by the carbothermal method, the NC layer alters the electronic structure of the Ni species through MSIs, which can be adjusted by controlling the thickness of the NC layer.

Accordingly, the NC layer with hydrophobic property not only enriches reactants and H_2_ in the microenvironment around the active sites but also influences mass transfer and adjust the electronic structure of the Ni species. These properties of the NC layer make SiO_2_@Ni@NC an excellent catalyst for the aqueous‐phase hydrogenation of vanillin at room temperature.

### 2.4 Effect of N‐Doping on Catalytic Performance for Aqueous‐Phase Hydrogenation

**Figure 6:**
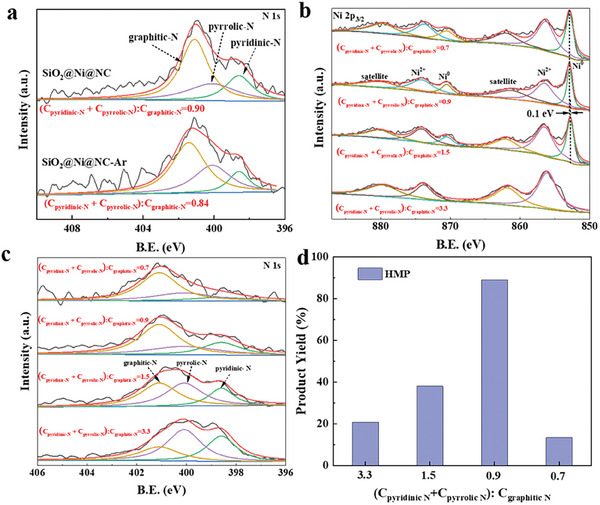
a) N 1s XPS spectra of SiO_2_@Ni@NC and SiO_2_@Ni@NC‐Ar; b) Ni 2p and c) N 1s XPS spectra of SiO_2_@Ni@NC catalysts with different (C_pyridinic‐N_ + C_pyrrolic‐N_):C_graphitic‐N_ ratios; d) HMP yields for vanillin hydrogenation over SiO_2_@Ni@NC catalysts with different (C_pyridinic‐N_ + C_pyrrolic‐N_):C_graphitic‐N_ ratios (25 °C, 2.0 MPa H_2_, 2 h, 1 mmol of vanillin, 10 mL of water, and 30 mg of catalyst).

Based on the promoting effect of the NC layer, we speculated that N doping in the NC layer might also play a role in catalytic hydrogenation. To clarify the role of N doping, we developed an approach to adjust the N content without changing the type of N species. Specifically, we replaced the carbothermic reduction atmosphere with inert Ar gas during the pretreatment process. XPS analysis (**Figure** **6**a) revealed that the composition of N species (pyridinic‐N, pyrrolic‐N, and graphitic‐N) on the surface of the Ni catalyst derived from the Ar treatment (denoted as SiO_2_@Ni@NC‐Ar) was similar to that of the catalyst derived from the N_2_ treatment (i.e., SiO_2_@Ni@NC), suggesting that the heat‐treating atmosphere has little influence on the N composition. However, the N content on the surface of these catalysts varied significantly. The SiO_2_@Ni@NC catalyst had a considerably higher N content than SiO_2_@Ni@NC‐Ar (Table S5, Supporting Information, entries 2 and 3), suggesting that N_2_ gas entered the carbon matrix to increase N doping in the carbon layer. Under the same reaction conditions, the catalytic activity of SiO_2_@Ni@NC was superior to that of SiO_2_@Ni@NC‐Ar for the aqueous‐phase hydrogenation of vanillin (Table S2, Supporting Information, entries 5 and 6). Similarly, SiO_2_@Ni@NC had a higher surface N content and exhibited superior activity compared with the SiO_2_@Ni@NC‐*x* samples with other NC layer thicknesses (Figure 5a; Table S5, Supporting Information). The increased N content can introduce additional active sites for catalytic reactions. These active sites can effectively adsorb reactants and catalyze the hydrogenation reaction, thereby enhancing the catalytic activity. Furthermore, N doping influenced the electronic structure of the Ni species (Figure 6b). Adjusting the charge distribution of the active metal can alter the electron density and thus influence the catalytic activity and selectivity.

In addition to the N content, the type of N species influenced the catalytic behavior of SiO_2_@Ni@NC during aqueous‐phase hydrogenation. In general, doped N exists in three main forms: pyridinic‐N, pyrrolic‐N, and graphitic‐N. In catalytic hydrogenation reactions, pyridinic‐N and pyrrolic‐N provide absorption sites for reactants and H_2_ molecules.^[^ ^46^ ^]^ Hence, we varied the carbothermic reduction temperature to adjust the proportion of these N species in the SiO_2_@Ni@NC catalyst. The ratios of pyridinic‐N and pyrrolic‐N to graphitic‐N ((C_pyridinic‐N_ + C_pyrrolic‐N_):C_graphitic‐N_) for the catalysts calcined at 500, 600, 700, and 800 °C under a N_2_ atmosphere were 3.3, 1.5, 0.9, and 0.7, respectively (Figure 6c). Notably, the absolute content of graphitic‐N was not significantly affected by the treatment temperature. Thus, the decrease in the (C_pyridinic‐N_ + C_pyrrolic‐N_):C_graphitic‐N_ ratio at higher temperatures was mainly caused by a decrease in the contents pyridinic‐N and pyrrolic‐N (Figure S18a, Supporting Information). For the aqueous‐phase hydrogenation of vanillin, the SiO_2_@Ni@NC catalyst with a (C_pyridinic‐N_ + C_pyrrolic‐N_):C_graphitic‐N_ ratio of 0.9 achieved an HMP yield of 89.2%, which was more than four‐, two‐, and sixfold those of the samples with ratios of 3.3, 1.5, and 0.7, respectively (Figure 6d). The XRD and XPS analyses (Figure 6b; Figure S18b, Supporting Information) revealed that the content of metallic Ni species in the SiO_2_@Ni@NC catalyst was inversely proportional to the (C_pyridinic‐N_ + C_pyrrolic‐N_):C_graphitic‐N_ ratio. Although high contents of pyridinic‐N and pyrrolic‐N provide more adsorption sites, they do not afford more active metals. Therefore, a balance must be established between active Ni and N species in the SiO_2_@Ni@NC catalyst to realize high activity. The catalyst prepared at 700 °C offered abundant adsorption and active sites, thus exhibiting superior catalytic performance for aqueous‐phase hydrogenation.

Overall, the type of N species and N content significantly influence the catalyst performance. With a similar proportion of N species, increasing in the content of doped N slightly can improve the catalyst performance. Therefore, the N content and proportion of N species can be adjusted to optimize the hydrogenation performance of a catalyst and enhance its application potential in related fields.

### 2.5 Theoretical Investigation of Vanillin Hydrogenation over SiO_2_@Ni@NC

**Figure 7:**
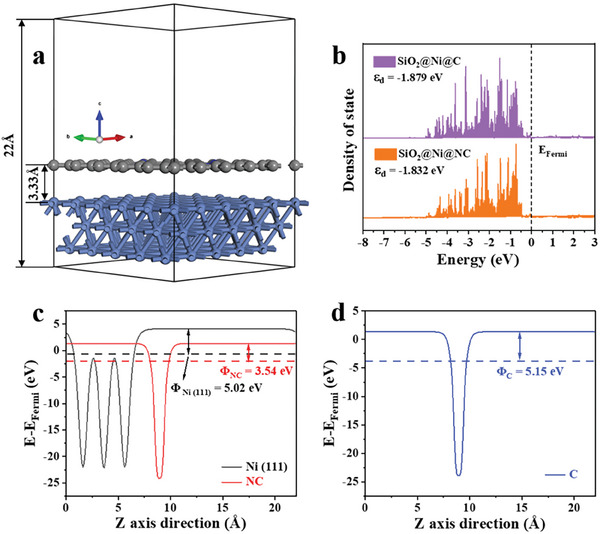
a) Schematic model of SiO_2_@Ni@NC; b) DOS of SiO_2_@Ni@C and SiO_2_@Ni@NC; work functions (*φ*) of c) Ni (111) and NC slabs and d) a carbon layer.

DFT calculations were conducted to elucidate the reaction mechanism and influence of the NC layer on catalytic performance. As shown in **Figure** **7**a, a theoretical model was constructed to simulate SiO_2_@Ni@NC. To determine the influence of N doping in graphitic carbon, models with undoped and optimally doped carbon were employed (SiO_2_@Ni@C and SiO_2_@Ni@NC, respectively; Figure S19, Supporting Information). Bader charge analysis was used to determine the amount of electron loss or gain for each C atom. As shown in Figure S20 (Supporting Information), 1.31 and 1.32 e^−^ were transferred to the N1 and N2 atoms, respectively, resulting in a positive charge on the neighboring atom and facilitating reactant adsorption.^[^ ^47^ ^]^ The interaction between NC and Ni also affects the d‐band center energy. Reduced empty d orbitals are known to be beneficial for electron transfer, and the catalytic activity of *d*‐block transition metals can be expressed by the *d*‐band center energy.^[^ ^48^ ^]^ The projected density of states (DOS) showed that the *d*‐band center energy was −1.879 eV for SiO_2_@Ni@C and −1.832 eV for SiO_2_@Ni@NC (Figure 7b). A *d*‐band center energy closer to the Fermi level (0 eV) facilitates the transfer of electrons from the catalyst surface to the adsorbate. Therefore, SiO_2_@Ni@NC is advantageous for vanillin adsorption and activation, consistent with the experimental results. The work function of a surface is a crucial parameter for investigating charge transfer at an interface. As shown in Figure 7c, the work functions (*φ*) of Ni (111) and NC were calculated to be 5.02 and 3.54 eV, respectively. Thus, electrons flow from NC to Ni until their Fermi energies reach the same level. In contrast, the work function of the system without N doping was 5.15 eV (Figure 7d), indicating that N doping changes the direction of electron flow at the interface between NC and Ni (111).

The catalytic performance of SiO_2_@Ni@NC was also assessed using DFT calculations. Because H_2_ is involved in vanillin conversion, we investigated the dissociative adsorption of H_2_ on the Ni (111) and SiO_2_@Ni@NC surfaces. The adsorption and dissociation configurations are shown in Figure S21 (Supporting Information). The adsorption energy of H_2_ on Ni (111) was 0.37 eV. The dissociation of H_2_ over Ni (111) was exothermic by 0.78 eV with a barrier of 0.12 eV. Furthermore, for the diffusion of H atoms on the Ni (111) surface, we computed a diffusion barrier of 0.16 eV between adjacent fcc site and hcp site. In contrast, hydrogen dissociation was spontaneous on the SiO_2_@Ni@NC surface (Supplementary Movie). Therefore, the NC layer can promote H_2_ dissociation and thus enhance the catalytic performance of SiO_2_@Ni@NC during hydrogenation.

**Figure 8:**
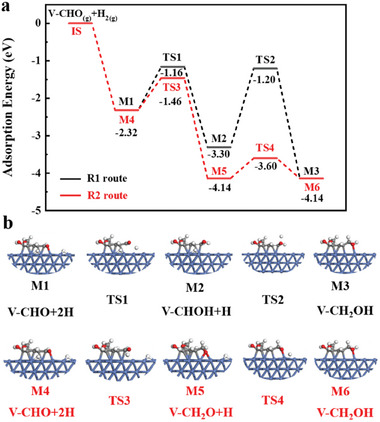
a) Proposed reaction mechanism for HMP formation on the Ni (111) surface; b) potential reaction pathways for vanillin conversion to HMP with optimized structures.

During vanillin hydrogenation, reactant adsorption on the catalyst surface is an important step. Therefore, different adsorption configurations were calculated for the NC and Ni (111) slab models (Figure S22 and Table S6, Supporting Information). Compared with the tilt and vertical adsorption models, the parallel adsorption modes exhibited more negative adsorption energies (−0.77 and −2.31 eV for NC and Ni (111), respectively). These results demonstrate that the parallel adsorption of vanillin on Ni (111) is energetically favorable and involves strong chemisorption. The parallel adsorption configuration promotes contact between the vanillin molecules and active sites on the catalyst surface, thereby improving the reaction rate and efficiency of carbonyl hydrogenation. Based on the most stable configuration, involving the parallel adsorption of vanillin, the complete reaction pathway for the conversion of vanillin (V‐CHO) to HMP (V‐CH_2_OH) over Ni (111) was calculated. The adsorption configurations and energies are shown in **Figure** **8** and Figures S23 and S24 and Table S7 (Supporting Information). Active H^*^ was integrated into a fcc or hcp site close to the substrate to search for the transition state. The conversion of V‐CHO to V‐CH_2_OH can occur via two competitive pathways (R1 and R2). In R1, the reaction is initiated by active H^*^ bonding to the oxygen atom of the C═O group, and this process is exothermic by 0.98 eV with a barrier of 1.16 eV (M1 to M2). The R2 pathway, which begins with the hydrogenation of the C atom in the C═O group, is also exothermic (1.82 eV) and the barrier is reduced to 0.86 eV (M4 to M5). Therefore, V‐CH_2_O formation via step 1 in R2 is both kinetically and thermodynamically favorable (Table S7, Supporting Information). In the subsequent step, active H^*^ attacks the alkoxyl intermediates (V‐CHOH and V‐CH_2_O), leading to the formation of V‐CH_2_OH. In the R1 pathway on Ni (111), this step is exothermic by 0.84 eV with a barrier of 2.10 eV (M2 to M3). However, in R2, the barrier is significantly reduced to 0.54 eV (M5 to M6). Accordingly, for hydrogenation of the C═O bond over the SiO_2_@Ni@NC catalyst, the R2 pathway is more favorable than the R1 pathway. The optimized hydrogenation pathway provides more precise and effective hydrogenation of the C═O bond under mild conditions.

**Figure 9:**
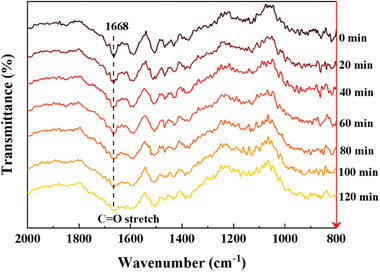
In situ IR spectra during vanillin hydrogenation over SiO_2_@Ni@NC.

HMP formation via vanillin hydrogenation was evidenced by in situ IR experiments, which monitored the catalytic performance of SiO_2_@Ni@NC at room temperature (**Figure** **9**). The catalyst and reactants were placed in an FTIR cell, H_2_ was introduced to remove air, and then the H_2_ pressure was maintained at 2 MPa. The conversion rate of vanillin was related to the reaction time. The characteristic carbonyl band of vanillin (*v*(C═O) at 1668 cm^−1^) gradually disappeared over 2 h, which was attributed to a decrease in the vanillin concentration. The SiO_2_@Ni@NC catalyst exhibited excellent hydrogenation performance at low temperatures, in good agreement with the catalytic results.

**Figure 10:**
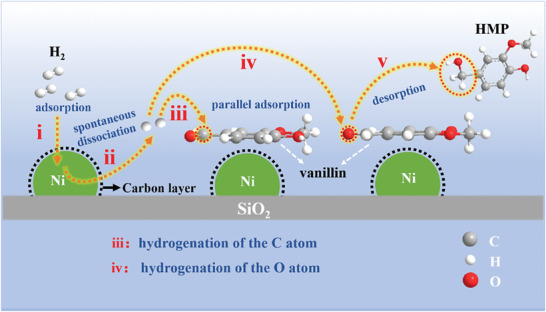
Schematic illustration of the hydrogenation mechanism over SiO_2_@Ni@NC.

Based on the above results, a mechanism for vanillin hydrogenation over SiO_2_@Ni@NC was proposed, as shown in **Figure** **10**. In the presence of the NC layer, H_2_ molecules are enriched around the active sites of the SiO_2_@Ni@NC catalyst. These molecules spontaneously dissociate into active H^*^ species, which preferentially hydrogenate the C atom in the C═O group of vanillin adsorbed parallel to the Ni (111) crystal plane. Subsequently, the O atom is hydrogenated to generate HMP, which is desorbed from the surface of the Ni (111) crystal plane.

### 2.6 Versatility of SiO_2_@Ni@NC for Hydrogenation

The hydrogenation ability of SiO_2_@Ni@C was evaluated using other unsaturated aldehyde ketones (**Table** **1**). The SiO_2_@Ni@NC catalyst effectively hydrogenated the model unsaturated compounds under mild reaction conditions. For example, 2‐methylbenzaldehyde was converted to 2‐methylbenzyl alcohol in 97.2% yield (Table 1, entry 1). Benzaldehyde was completely converted to benzyl alcohol near room temperature (Table 1, entry 2), indicating the excellent performance of SiO_2_@Ni@NC for the hydrogenation of C═O bonds in aromatic aldehyde compounds. In addition, the SiO_2_@Ni@NC catalyst also converted other furan compounds, such as furfural, 5‐methylfurfural, and 5‐hydroxymethylfurfural, into the corresponding alcohols in good yields (Table 1, entries 3–5). Thus, SiO_2_@Ni@NC is a versatile catalyst for the aqueous‐phase hydrogenation of various unsaturated aldehydes and ketones.

**Table 1:** Hydrogenation of various unsaturated aldehydes and ketones over the SiO_2_@Ni@NC catalyst^a)^.

Entry	Substrate	Product	Temperature [°C]	Time [h]	Yield [%]
1	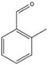	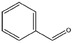	60	2	97.2
2	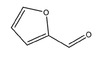	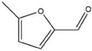	40	2	99.9
3	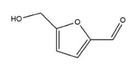	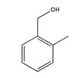	60	2	92.5
4	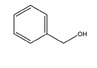	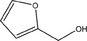	80	2	98.6
5	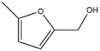	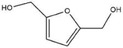	40	4	81.2

^a)^ Reaction conditions: 2 MPa H_2_, 1.0 mmol of substrate, 10 mL of water, and 30 mg of catalyst.

## 3 Conclusion

In summary, we prepared an efficient amphiphilic catalyst with SiO_2_ as a hydrophilic core and NC as a hydrophobic surface. The as‐prepared SiO_2_@Ni@NC catalyst exhibited high catalytic performance for the aqueous‐phase conversion of vanillin at room temperature, achieving ≈100% conversion to HMP and excellent reusability over five cycles. The high activity of SiO_2_@Ni@NC was attributed to the synergistic effect of the amphiphilic support and encapsulated Ni NPs. Specifically, the hydrophilic core effectively promoted catalyst dispersion in the aqueous phase, whereas the hydrophobic NC layer protected the highly active Ni NPs from leaching or oxidation and regulated the electron density of Ni via MSIs. Moreover, experimental results and DFT calculations revealed that NC facilitated the adsorption of vanillin molecules and the spontaneous dissociation of hydrogen to promote aqueous‐phase hydrogenation. This encapsulated catalyst, with enhanced activity and stability, provides a new approach for biomass transformation.

## Conflict of Interest

The authors declare no conflict of interest.

## Supporting information

Supporting Information

Supplemental Movie 1

## Data Availability

The data that support the findings of this study are available from the corresponding author upon reasonable request.
