# Supplementary material for: Toward High‐Performance Hydrogenation at Room Temperature Through Tailoring Nickel Catalysts Stable in Aqueous Solution
Source: Adv Sci (Weinh). 2024 Apr 6;11(24):2309303. doi: 10.1002/advs.202309303 (PMC11199984; doi:10.1002/advs.202309303)
Supplement: Supplementary file 1 — Supporting Information [file ADVS-11-2309303-s001.docx]

**Supporting Information**

**Towards High-Performance Hydrogenation at Room Temperature through Tailoring Nickel Catalysts Stable in Aqueous Solution**

*Zidan Zou,^a,b^ Yue Shen,^a,b^ Xiao Zhang,^a^ Wenchao Li,^a^ Chun Chen,^*a,b^ Diancai Fan,^c^ Haimin Zhang,^a,b^ Huijun Zhao,^a,d^  Guozhong Wang^*a,b^*

*^a^* Key Laboratory of Materials Physics, Centre for Environmental and Energy Nanomaterials, Key Laboratory of Materials Physics, Institute of Solid State Physics, HFIPS, Chinese Academy of Sciences, Hefei, 230031, China.

*^b^* Science Island Branch, Graduate School of USTC, Hefei, 230026, China.

*^c^* Anhui Haoyuan Chemical Group Co., Ltd. Fuyang, 236056, China.

*^d^* Centre for Clean Environment and Energy, Gold Coast Campus, Griffith University, Queensland 4222, Australia.

E-mail: chenchun2013@issp.ac.cn (C.C), gzhwang@issp.ac.cn (G.W).

**Contents**

**1. Experimental Sections and Characterizations**

**1.1 Chemicals and reagents**

**1.2 Synthesis of catalysts**

**1.3 Characterization**

**2. Catalytic performance tests**

**3. Computational details**

**4. Supplementary Figures and Tables**

**1. Experimental Sections** **and Characterizations**

**1.1 Chemicals and reagents**

Ammonia (NH_3_·H_2_O), formaldehyde aqueous solution, anhydrous ethanol, methanol, and 2-propanol were purchased from Sinopharm Chemical Reagent Co. Ltd. resorcinol, nickel nitrate hexahydrate (Ni(NO_3_)_2_·6H_2_O), n-butanol, vanillin, 2-methylbenzaldehyde, benzaldehyde, furfural, 5-methylfurfural and 5-hydroxymethylfurfural were purchased from Aladdin Reagent Company. Deionized water with a specific resistance larger than 18 MΩ was used throughout the experiments.

**1.2 Synthesis of catalysts**

**Preparation of SiO_2_@Ni(OH)_2_ nanospheres:** The core-shell structure SiO_2_@Ni(OH)_2_ was first synthesized by a modified hydrothermal method. Specifically, silica spheres (0.32 g) were homogeneously dispersed into 20 mL deionized water and then added to a solution of 0.436 g Ni(NO_3_)_2_·6H_2_O and 3 mL NH_3_∙H_2_O in deionized water (30 mL) with magnetic stirring. After that, the homogeneous solution was transferred into an autoclave (100 mL) and heated at 90 ℃ for 12 h. The light green precipitate was washed repeatedly with deionized water and ethanol until the pH value was 7. The precipitate after centrifugation was dried in an oven at 60 ℃ to obtain the hierarchical configuration precursor with a core-shell structure (SiO_2_@Ni(OH)_2_).

**Preparation of SiO_2_@Ni@NC:** The core-shell structure SiO_2_@Ni@NC was synthesized by precipitation method. In detail, 0.2 g SiO_2_@Ni(OH)_2_ was dispersed in 70 mL anhydrous ethanol and 10 mL H_2_O, after mixing evenly, 1 mL ammonia solution was added into above solution quickly and then stirred for 10 min. After obtaining a uniform mixture, a certain amount of resorcinol and formaldehyde aqueous solution were added into above and keep stirring for another 24 h. The products were separated by centrifugation, washed with water and ethanol several times, dried in oven, and treated under nitrogen atmosphere at 700 ℃ for 5 h with a heating rate of 2 ℃ min^−1^ to obtain the encapsulated Ni-based amphiphilic catalyst (SiO_2_@Ni@NC).

**Preparation of SiO_2_@Ni**: The SiO_2_@Ni(OH)_2_ was treated in H_2_/Ar (10 vol%) at 700 ℃ for 5 h with a heating rate of 2 ℃ min^−1^ to obtain the SiO_2_@Ni catalyst.

**1.3 Characterization**

The crystalline structures of the materials were confirmed through Powder X-ray diffraction (XRD, Philips X’ pert PRO) within the 2θ range of 10°~90° using a copper target (Cu Kα, λ= 0.15432 nm) at 40 kV and 40 mA. The morphology and the structure of the synthesized materials was observed by the field emission scanning electron microscopy (FESEM, SU8020) and transmission electron microscopy (TEM, JEOL-2010) with an energy dispersive X-ray spectrometer (EDS Oxford, Link ISIS). The surface element information of the synthesized materials was investigated through XPS analysis, which was executed on an ESCALAB 250 X-ray photoelectron spectrometer (Thermo, U.S.A.) with Al Kα_1,2_ monochromatized radiation at 1486.6 eV X-ray source. The Ni contents of the materials were determined by inductively coupled plasma-atomic emission spectrometer (ICP-AES). Temperature-programmed-desorption with H_2_ (H_2_-TPD) was carried out by a Micromeritics AutoChem II 2920 instrument equipped with a thermal conductivity detector (TCD). The surface area and pore size distribution of the material were measured by the nitrogen adsorption experiment, which was run at −196 ℃ on a Micrometrics ASAP 2020M. Raman spectra were obtained by LabRAM HR800 confocal microscope Raman system (Horiba Jobin Yvon) using an Ar ion laser operating at 532 nm. The in situ FTIR was performed using an INVENIOR FT-IR (Bruker) spectrometer equipped with an MCT detector. The concentration change of vanillin in solution was determined by UV-vis 2700 spectrophotometer (Shimadzu, Japan). And the ^1^H NMR spectroscopy measurement (Bruker AVANCE AV III 400) was used for structural analysis of product. Elemental analysis was conducted using an elemental analyzer (Elementar UNICUBE).

**2. Catalytic performance tests**

The hydrogenation reaction was carried out using a 25 mL stainless steel autoclave equipped with a mechanical stirrer (Anhui Kemi Machinery Technology Co. Ltd). Typically, 30 mg of SiO_2_@Ni@NC catalyst and 1 mmol of reactant were dispersed into 10 mL H_2_O or other solvents by ultrasound for a while, and then transferred to the reactor. After assembling the reactor, it was purged with N_2_ multiple times to purge the air inside, then pressurized with a certain amount of H_2_ under the condition that there was no gas leakage issue in the reactor. After that, the reactor was heated to the desired temperature and kept for a specified time with a stirring rate of 600 rpm. When the reaction finished, the reactor was cooled quickly to room temperature, and then the product and catalyst were filtered out with a 0.22 μm filter. Finally, the filtered product was extracted with ethyl acetate (3 mL × 3 times) to gather the organic phase.

The conversion rate and product selectivity of vanillin were calculated by the following formula:

 (1)

 (2)

The formula for calculating the TOF was as follows:

 (3)

Here, *n*_vanillin_ and *n*_Ni_ refer to the vanillin and Ni moles, respectively. *D* is the dispersion of Ni and *t* is the reaction time in hours.

**3. Computational details**

We have employed the Vienna Ab Initio Package (VASP)^[1]^ to perform all the spin-polarized density functional theory (DFT) calculations within the generalized gradient approximation (GGA) using the Perdew-Burke Ernzerhof (PBE) formulation.^[2]^ The projected augmented wave (PAW) potentials^[3]^ were chosen to describe the ionic cores and take valence electrons into account using a plane wave basis set with a kinetic energy cutoff of 400 eV. The D3 correction method (DFT-D3) was employed in order to include van der Waals (vdW) interactions.^[4]^ The subsequent VASP calculation data results were processed using the VASPKIT code.^[5]^ The lattice mismatch of the heterojunction SiO_2_@Ni@NC to be as small as 1.0 %. The conjugate gradient algorithm was used in geometry optimization calculations. Optimized structures were obtained by minimizing the forces on each ion until they were less than 0.02 eV/Å. The energy convergence criteria set to 10^-5^ eV. The gamma (Γ) centered 2 × 2 × 1 Monkhorst-Pack k-point sampling was conducted. The (5 × 5) Ni (111) supercell was constructed. The top two atomic layers of the surface were allowed to relax together with the molecule adsorbate, while the bottom layer was kept fixed to present the bulk properties. To minimize the interlayer interactions, the vacuum distance was larger than 20 Å. Transition states (TSs) for the elementary reactions were located using the climbing-image nudged elastic band (CI-NEB) method^[6-7]^, and were further optimized using the dimer method with an energy convergence criteria of 10^-7^ eV. The optimized transition states were further verified as having a single imaginary frequency in the direction of the reaction coordinate. The Hessian matrices for the frequency calculations were determined using the finite displacement scheme with a step size of 0.015 Å. Bader charge analysis was performed to discuss the electronic properties.^[8]^

The activation energy barrier (*E*_a_) and reaction energy (*E*_r_) were calculated by using eqs 4 and 5, where *E*_IS_, *E*_TS_, and *E*_FS_ are the energies of the corresponding initial state (IS), transition state (TS), and the final state (FS), respectively.

*E*_a_ = *E*_TS_ – *E*_IS_ (4)

*E*_r_ = *E*_FS_ – *E*_IS_ (5)

The adsorption energy (E_ads_) was calculated by the following equation:

*E*_ads_ = *E*_(adsorbate + surface)_ – *E*_(adsorbate)_ – *E*_(surface)_ (6)

Where *E*_(adsorbate + surface)_ is the total energy of the adsorbate interacting with the surface; *E*_(adsorbate)_ and *E*_(surface)_ are the energies of the free adsorbate in the gas phase and the bare surface, respectively. A negative value corresponds to exothermic adsorption, with more negative values corresponding to stronger adsorption.

**4. Supplementary Figures and Tables**


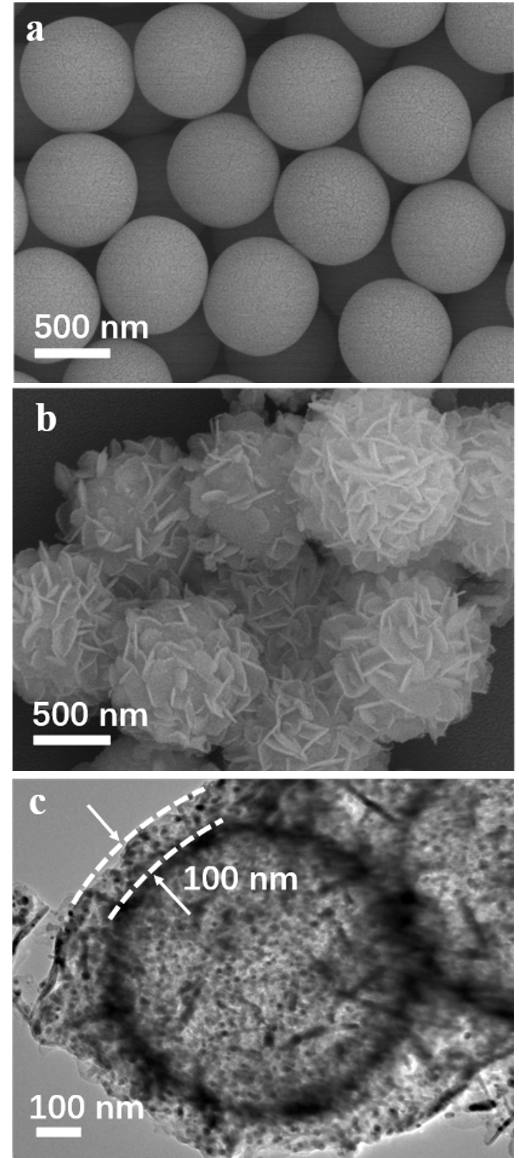


**Figure S1.** (a) SEM spectra of SiO_2_ nanospheres; (b) SEM spectra of SiO_2_@Ni(OH)_2_@RF; (c) TEM spectra of etched SiO_2_@Ni.


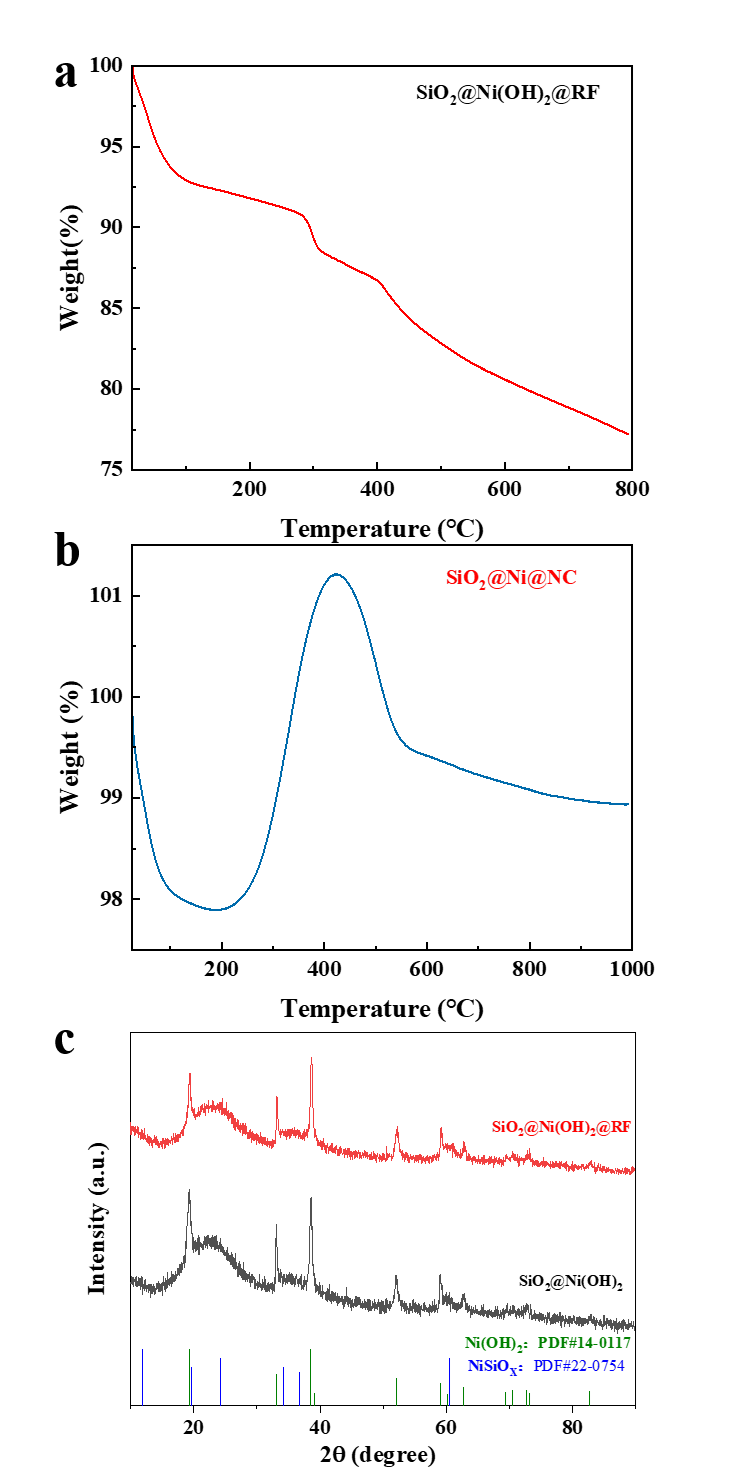


**Figure S2.** TG curve of (a) SiO_2_@Ni(OH)_2_@RF at N_2_ atmosphere and (b) SiO_2_@Ni@NC in air; (c) XRD patterns of SiO_2_@Ni(OH)_2_@RF and SiO_2_@Ni(OH)_2_.


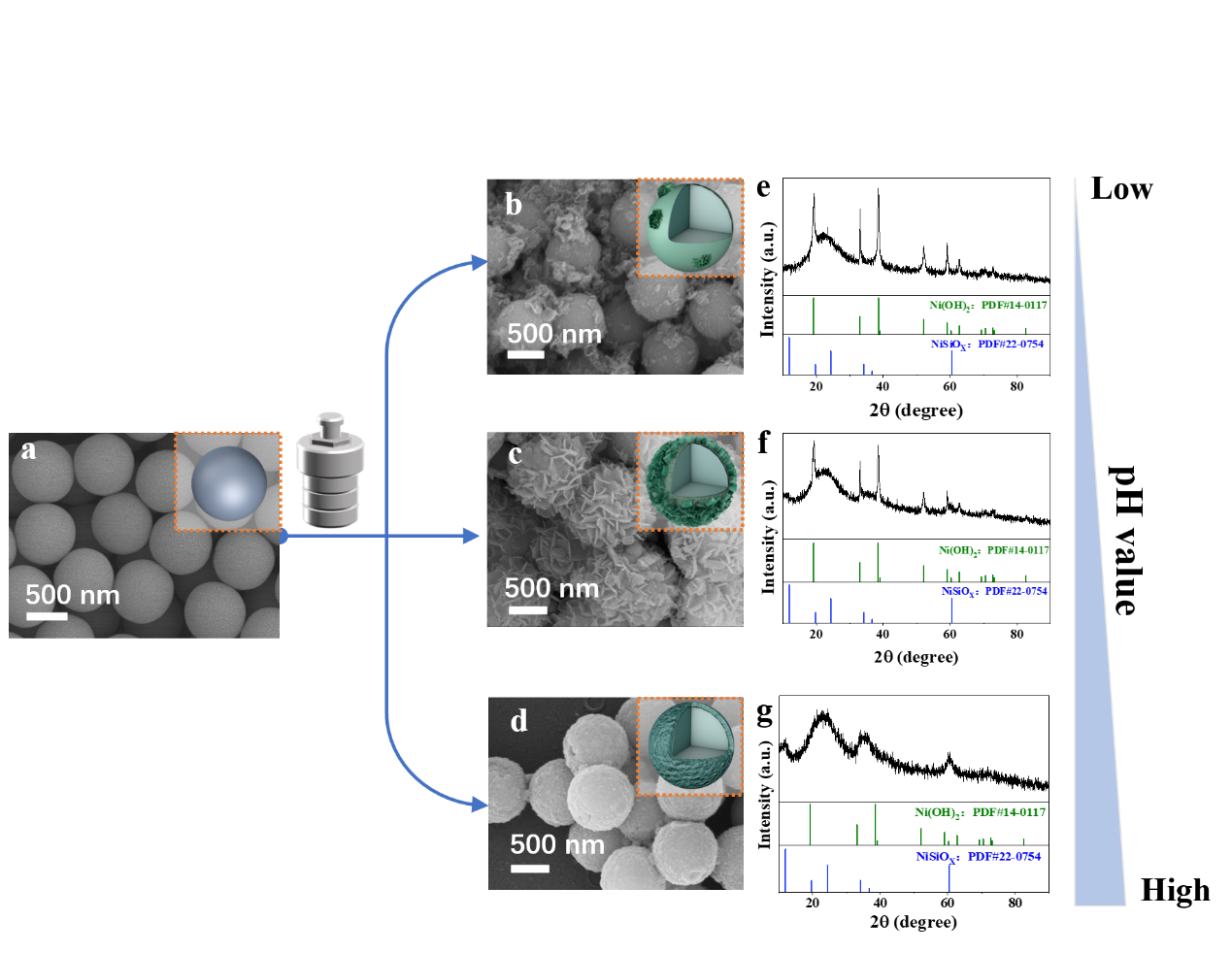


**Figure S3.** (a) SEM spectra of SiO_2_ nanospheres; (b-d) SEM spectra of obtained SiO_2_@Ni(OH)_2_ samples after adjusting pH value; (e-g) The corresponding XRD patterns of obtained SiO_2_@Ni(OH)_2_ samples after adjusting pH value.


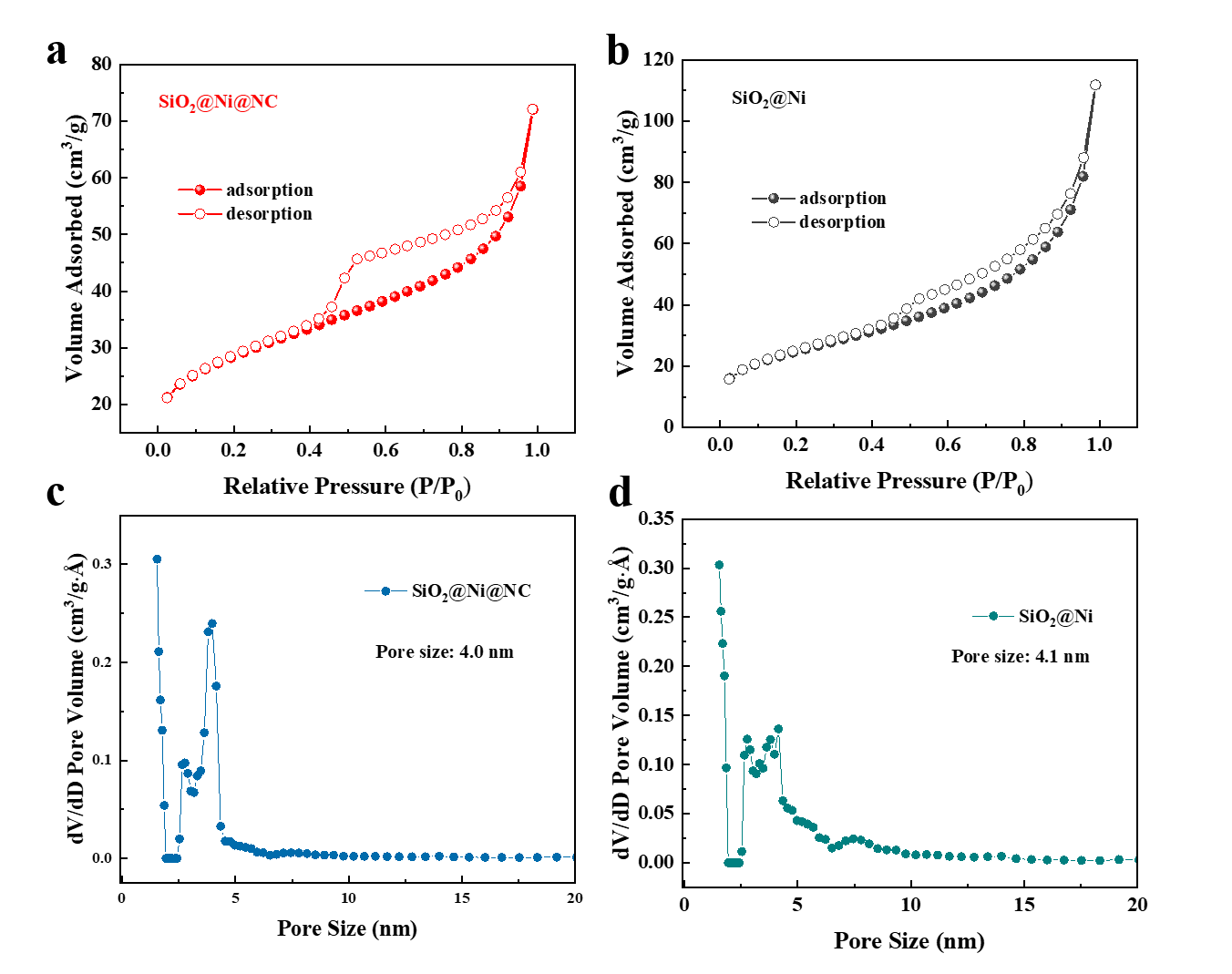


**Figure S4.** N_2_-adsorption/desorption isotherms of (a) SiO_2_@Ni@NC and (b) SiO_2_@Ni; the pore size distribution of (c) SiO_2_@Ni@NC and (d) SiO_2_@Ni.


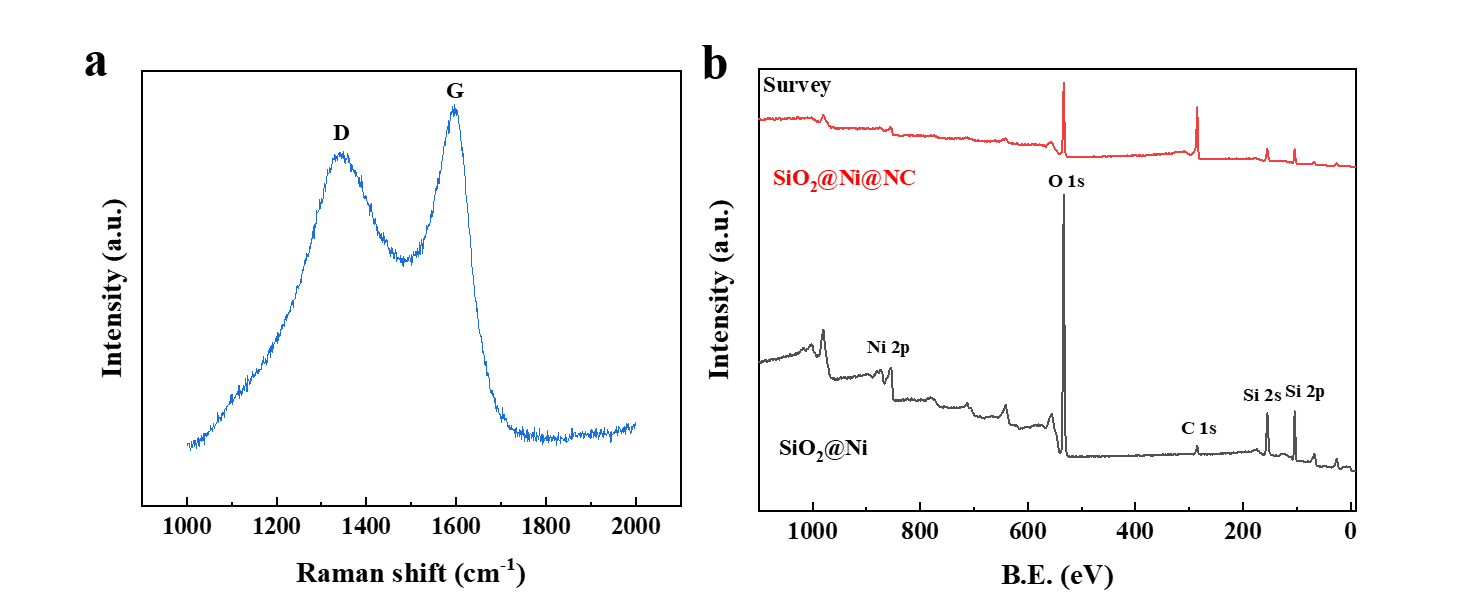


**Figure S5.** (a) Raman spectra of SiO_2_@Ni@NC; (b) XPS survey spectra of SiO_2_@Ni@NC and SiO_2_@Ni.


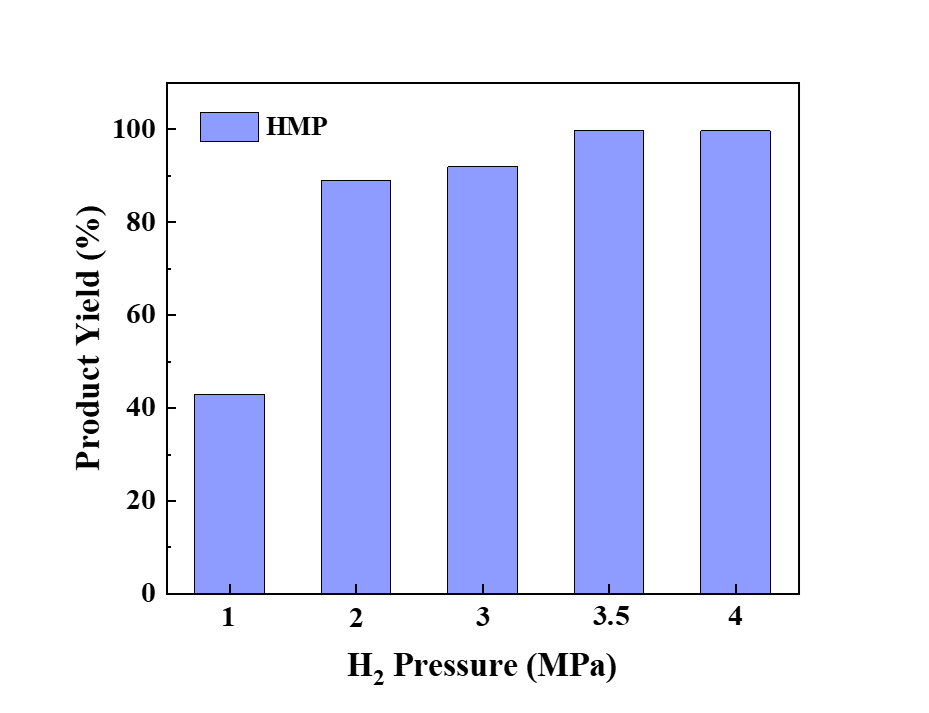


**Figure S6**. The effect of H_2_ pressure in hydrogenation of vanillin over the SiO_2_@Ni@NC (25 ℃, 2 h, 1 mmol vanillin, 10 mL water, and 30 mg catalyst).

The effect of H_2_ pressure on activity and product distribution was investigated at 25 ℃. With the increase of hydrogen pressure, the conversion of vanillin gradually increases, because increasing hydrogen pressure would increase the concentration of hydrogen on the catalyst surface, thereby accelerate the reaction rate. At extremely low hydrogen pressure (1 MPa), the SiO_2_@Ni@NC catalyst can solely convert 43.3% vanillin to HMP. When increases the hydrogen pressure to 2 MPa, the reaction rate of hydrogenation increases significantly and is close to completely conversion with HMP as the sole product.

The results demonstrate that the design catalyst can convert vanillin to sole product of HMP under room temperature even at a high H_2_ pressure. The high activity of Ni combined with appropriate regulation of catalyst structure enables the SiO_2_@Ni@NC catalyst to exhibit excellent activity for C=O activation and hydrogenation, and can completely convert vanillin to HMP in the aqueous phase at room temperature.


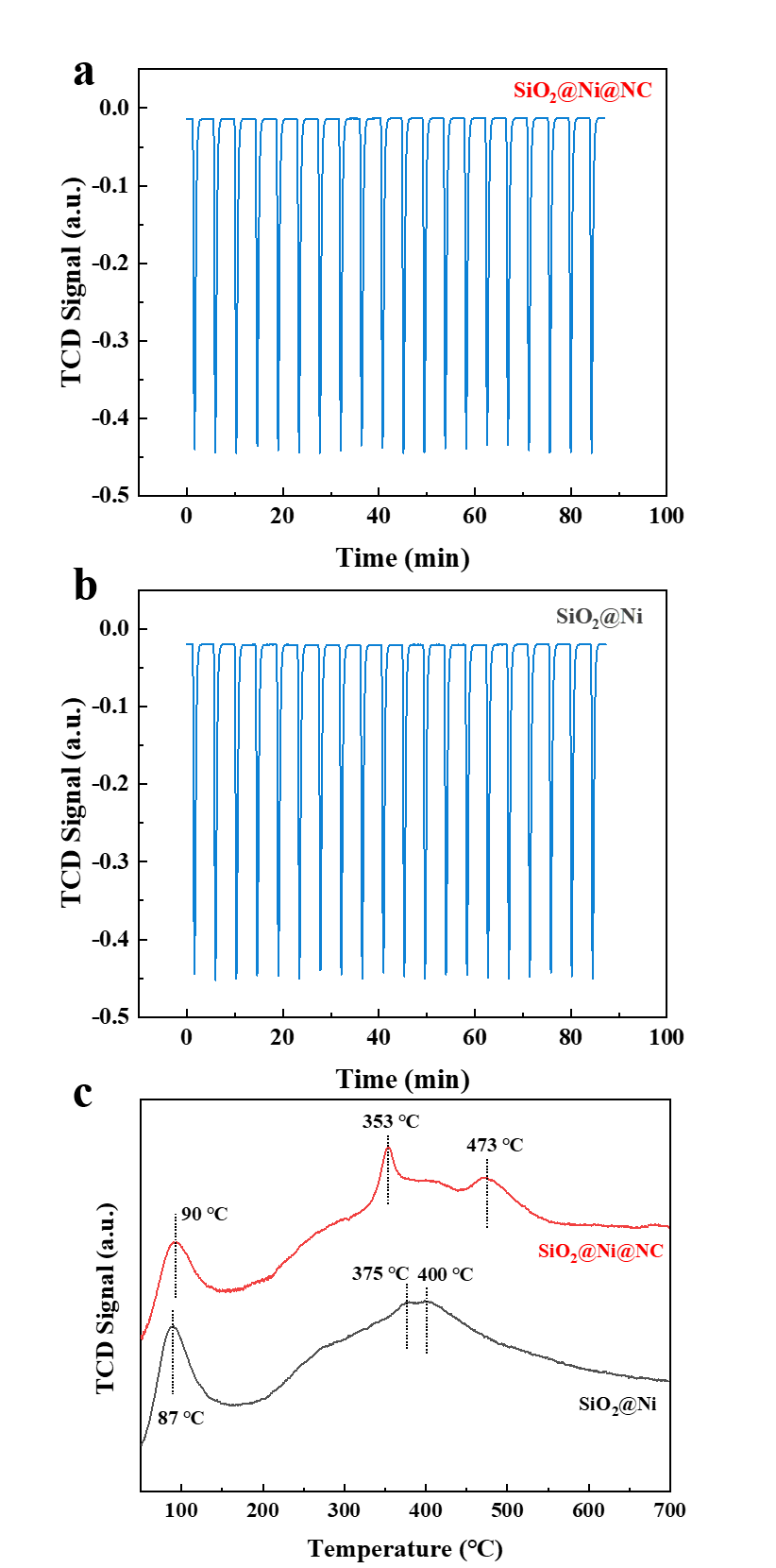


**Figure S7.** CO-TPD of (a) SiO_2_@Ni@NC and (b) SiO_2_@Ni; (c) H_2_-TPD of SiO_2_@Ni@NC and SiO_2_@Ni.


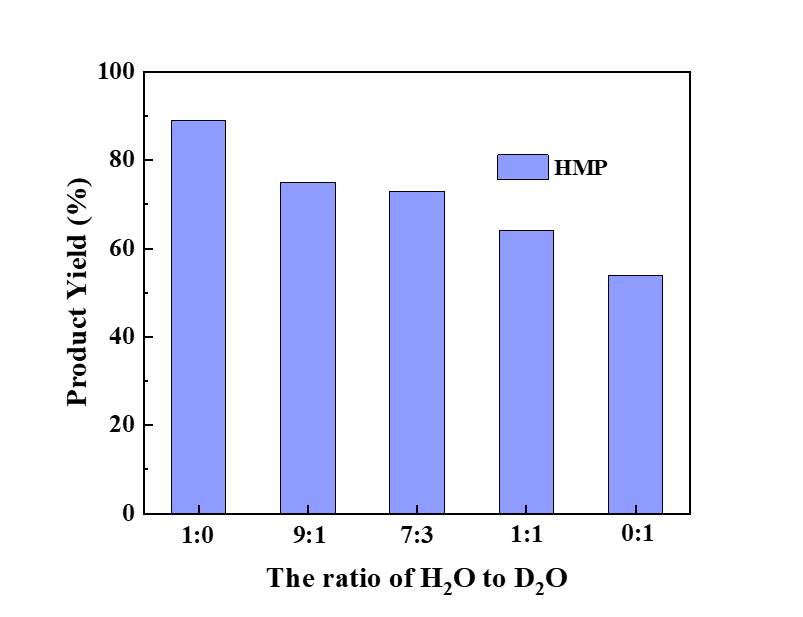


**Figure S8.** Effect of water and deuteroxide volume ratios in hydrogenation of vanillin over SiO_2_@Ni@NC (25 ℃, 2.0 MPa H_2_, 2 h, 1 mmol vanillin, 10 mL solvent, and 30 mg catalyst).


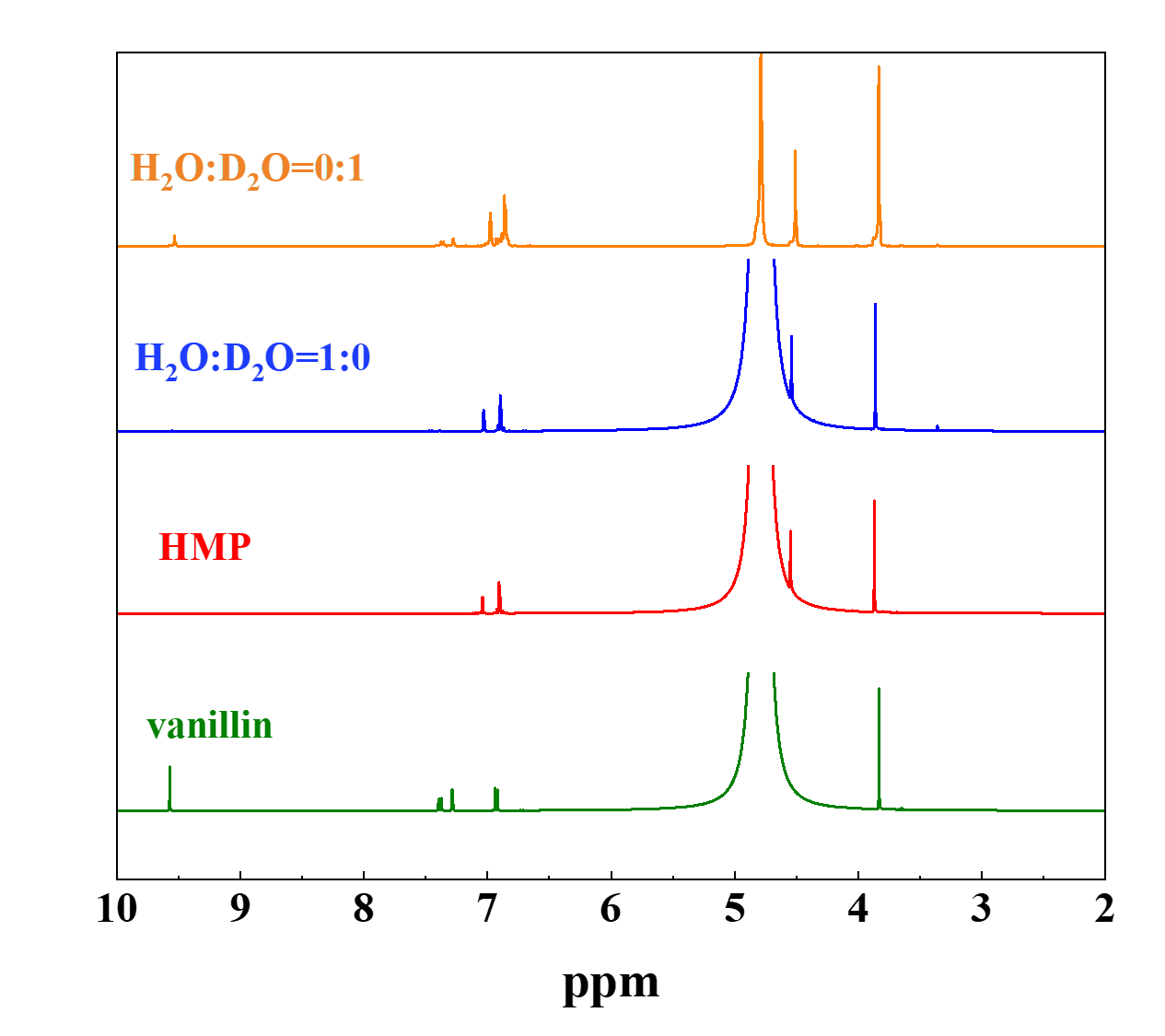


**Figure S9.** The 400 MHz ^1^H NMR spectrums of vanillin, HMP, and the hydrogenated product in different volume ratios of water and deuteroxide.


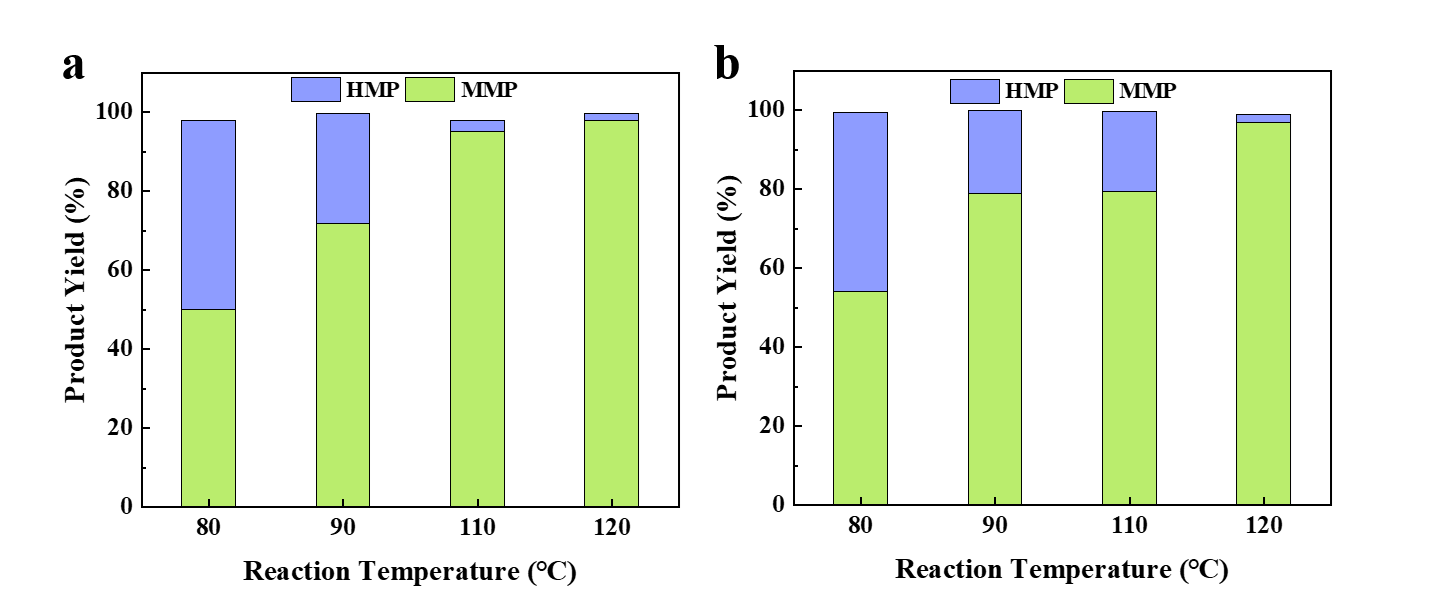


**Figure S10**. The product yields of vanillin hydrogenation and selectivity to HMP or MMP in different reaction temperatures over the (a) SiO_2_@Ni@NC and (b) SiO_2_@Ni (2.0 MPa H_2_, 1 mmol vanillin, 10 mL water, 6 h, and 30 mg catalyst).


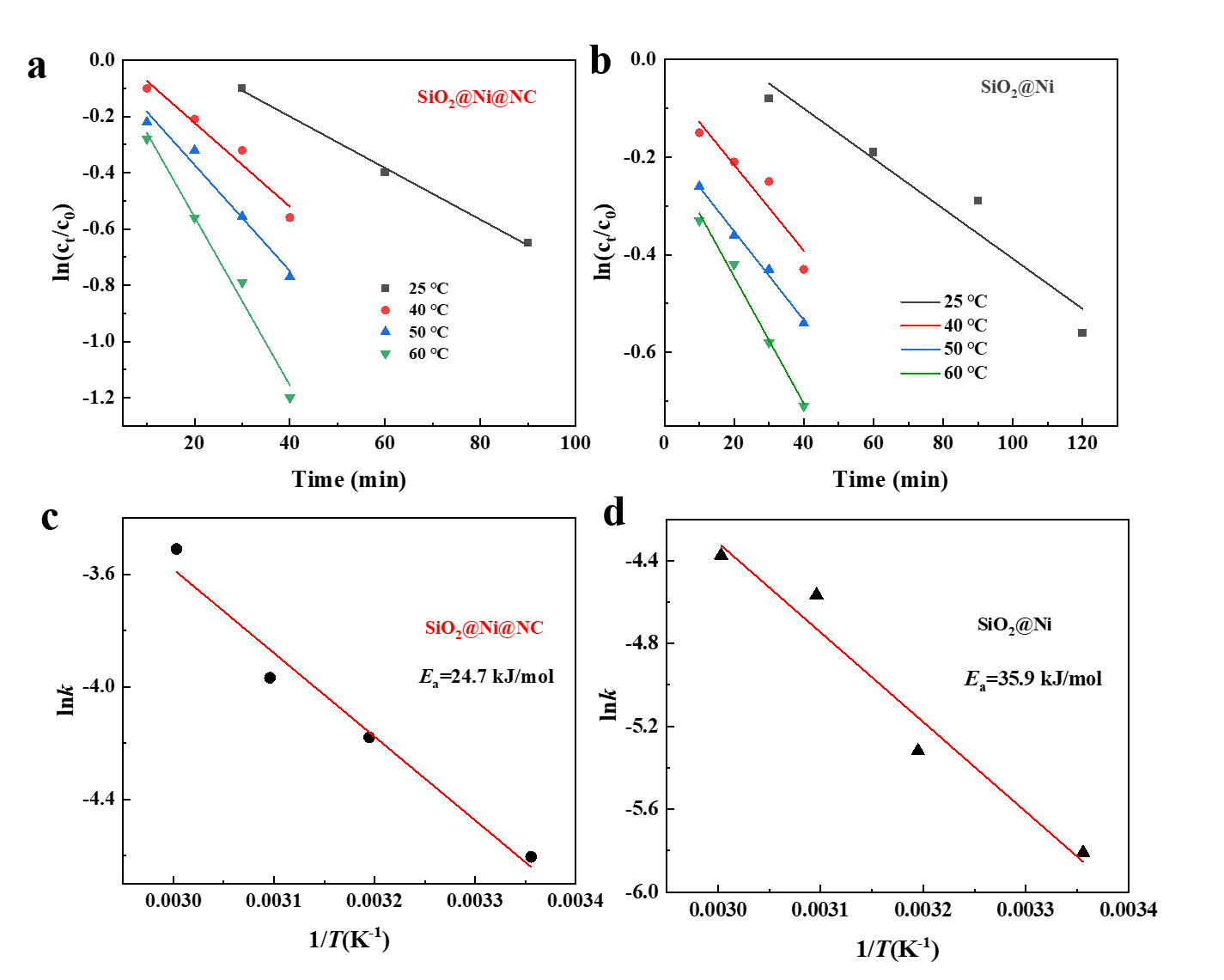


**Figure S11**. Kinetic profile of vanillin hydrogenation by (a) SiO_2_@Ni@NC and (b) SiO_2_@Ni; the Arrhenius plots for hydrogenation of vanillin by (c) SiO_2_@Ni@NC and (d) SiO_2_@Ni.

The detailed kinetics experiments have been carried out to further explore the catalytic reaction rate and catalytic activity and corresponding results are shown in Figure S11. The reactant conversion as a function of reaction time at the different reaction temperatures. On these bases, the vanillin hydrogenation exhibits first-order reaction kinetic behaviors from 25 ℃ to 60 ℃, as shown in a linear relation between Napierian logarithm of conversion (ln (1-conversion of reactant)) and reaction time. The slop of the linear curve is the reaction rate constant *k* (min^−1^). It can be seen that the rate of vanillin hydrogenation by SiO_2_@Ni@NC is faster than that of SiO_2_@Ni. The reaction activation energy (*E*_a_) is further calculated based on the Arrhenius equation (ln*k*=-*E*_a_/RT + C), and the values are also listed in Figure S11c,d. The reaction of vanillin hydrogenation by SiO_2_@Ni@NC shows a lower *E*_a_ value (24.7 kJ/mol) compared with the SiO_2_@Ni (35.9 kJ/mol), suggesting that the SiO_2_@Ni@NC catalyst has better hydrogenation performance than the SiO_2_@Ni catalyst at low temperatures, which is in accordance with other experiments result.


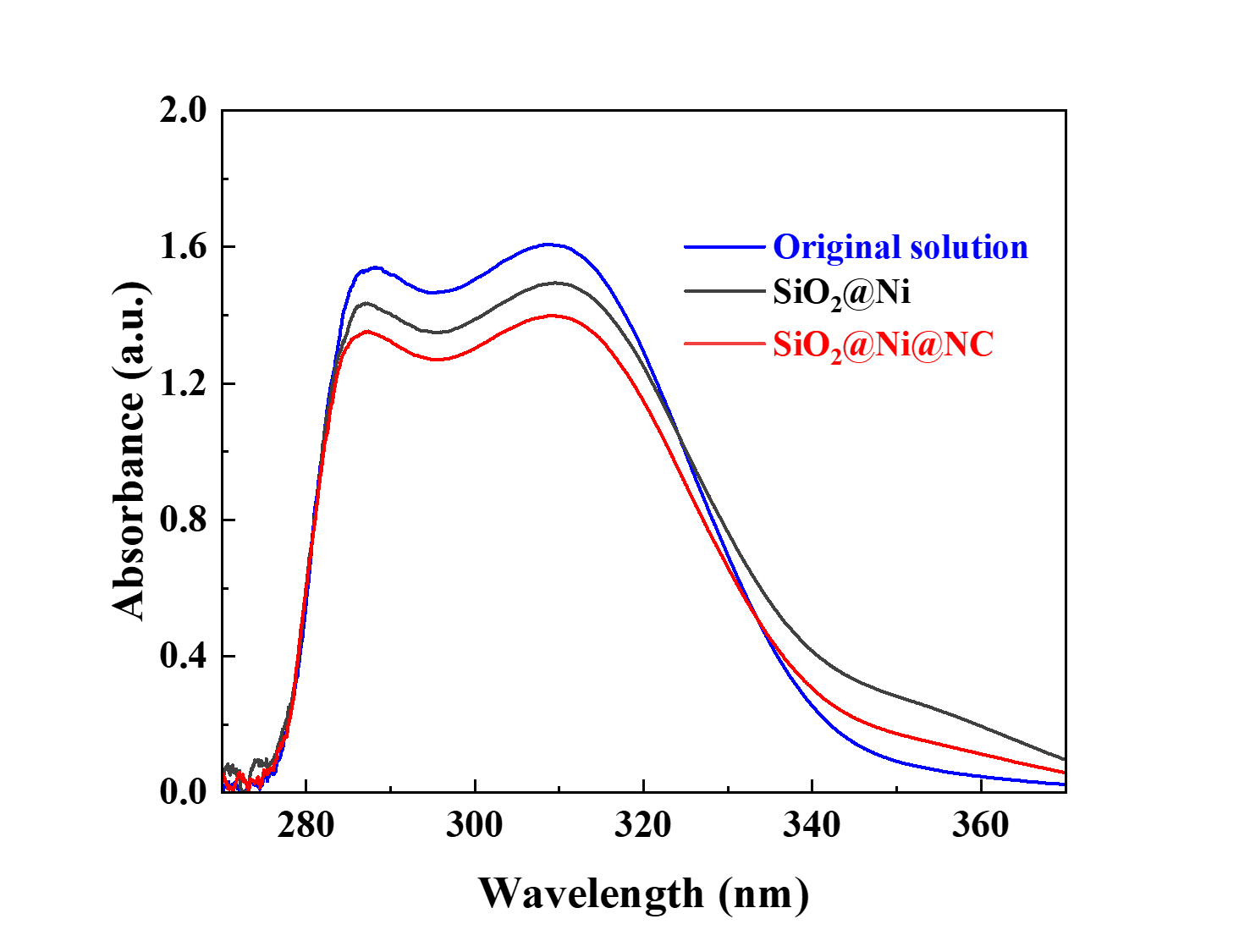


**Figure S12.** UV-vis spectrums of vanillin in the solution after adsorption on SiO_2_@Ni@NC and SiO_2_@Ni during 30 min.


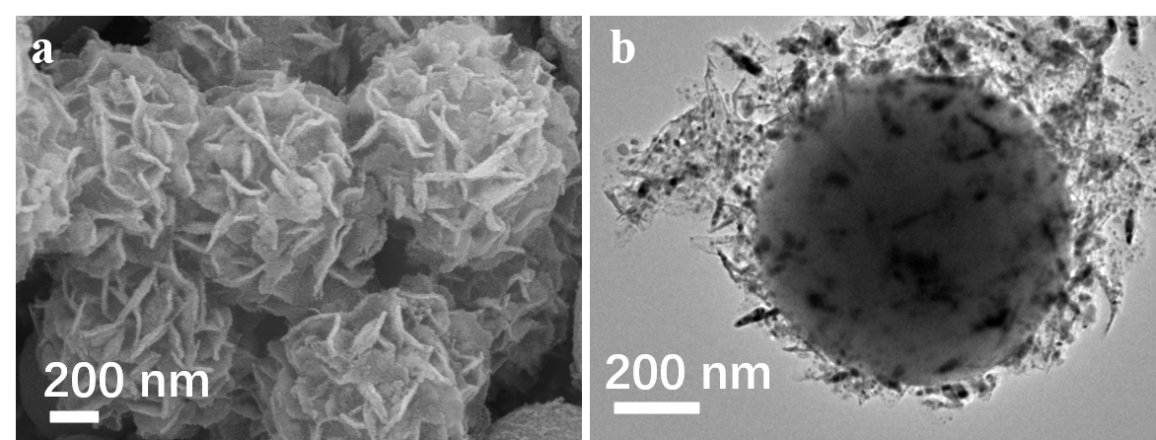


**Figure S13.** (a) SEM and (b) TEM images of the reused SiO_2_@Ni@NC.


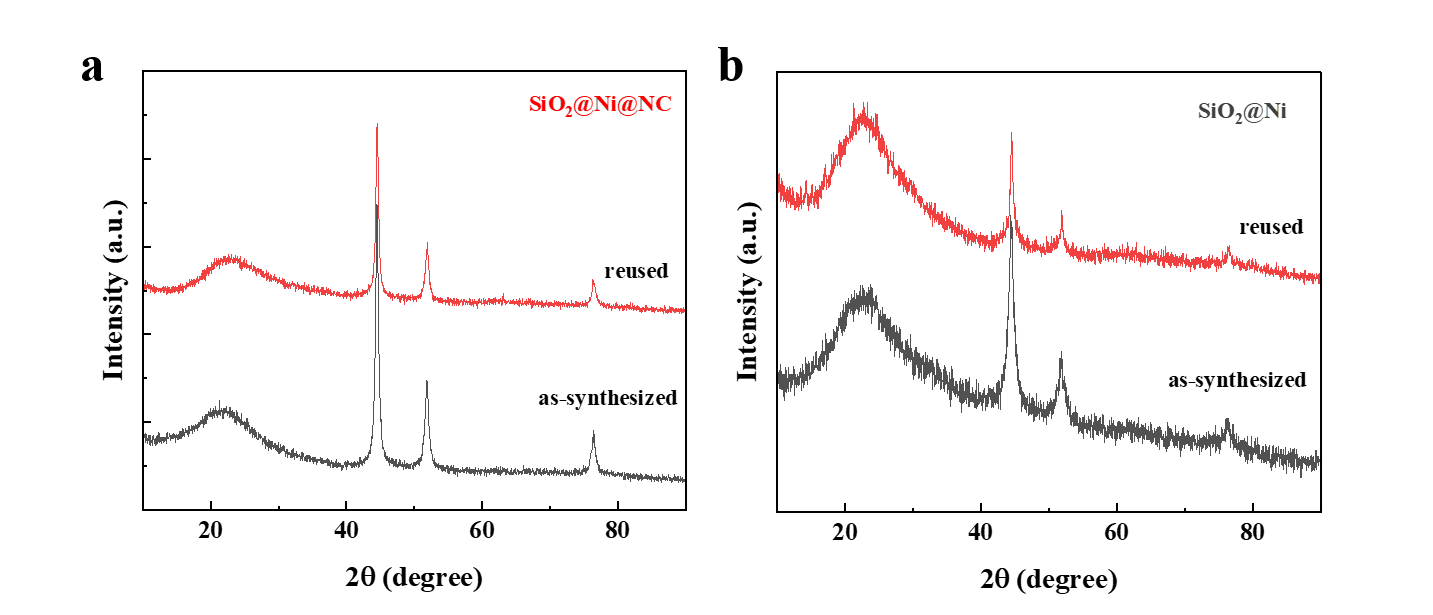


**Figure S14.** XRD patterns of the as-synthesized and reused (a) SiO_2_@Ni@NC and (b) SiO_2_@Ni.


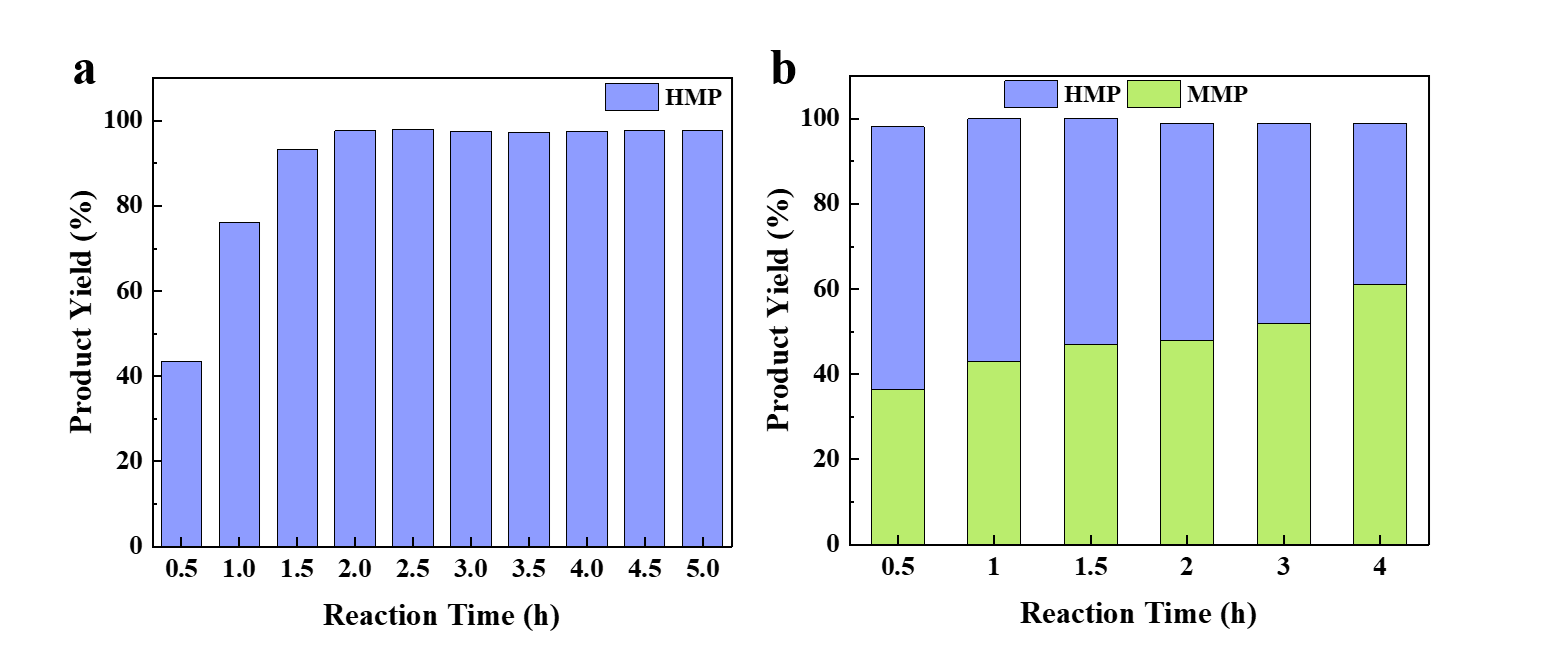


**Figure S15**. The gram-scale experiment for hydrogenation of vanillin by the SiO_2_@Ni@NC at (a) 50 ℃ and (b) 90 ℃. (Reaction conditions: 300 mg catalysts, 1.52 g (10 mmol) vanillin, 2 MPa H_2_, 100 ml H_2_O).

The large-scale experiment was carried out in a 250 mL stainless steel autoclave equipped with a mechanical stirrer. At the reaction temperature of 50 ℃, only HMP was produced, and with the extension of reaction time, the yield of HMP gradually increased, reaching 100% within 2 h. When the reaction temperature elevated to 90 ℃, 36.7% of MMP is generated within 0.5 h, and the yield of MMP further achieved 61.7% in 4 h. These results indicate that even if the reaction system is magnified tenfold, the SiO_2_@Ni@NC catalyst still exhibits excellent hydrogenation and HDO performance.


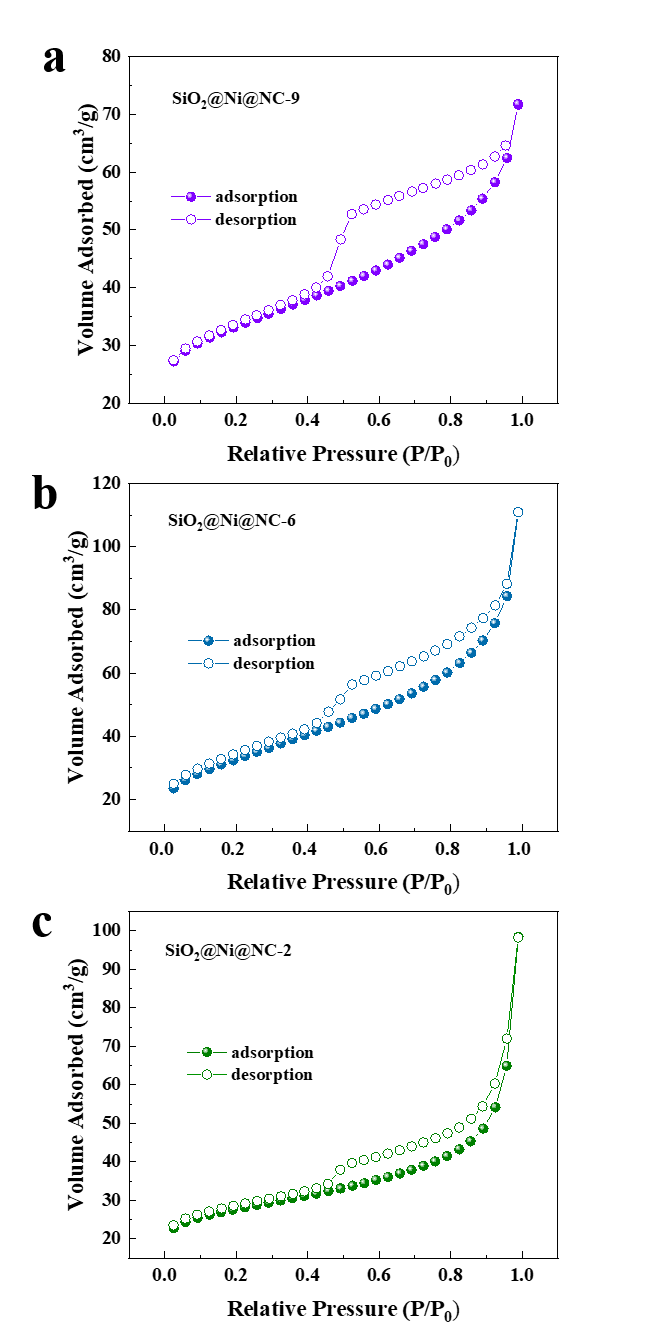


**Figure S16.** N_2_-adsorption/desorption isotherms of (a) SiO_2_@Ni@NC-9, (b) SiO_2_@Ni@NC-6 and (c) SiO_2_@Ni@NC-2.


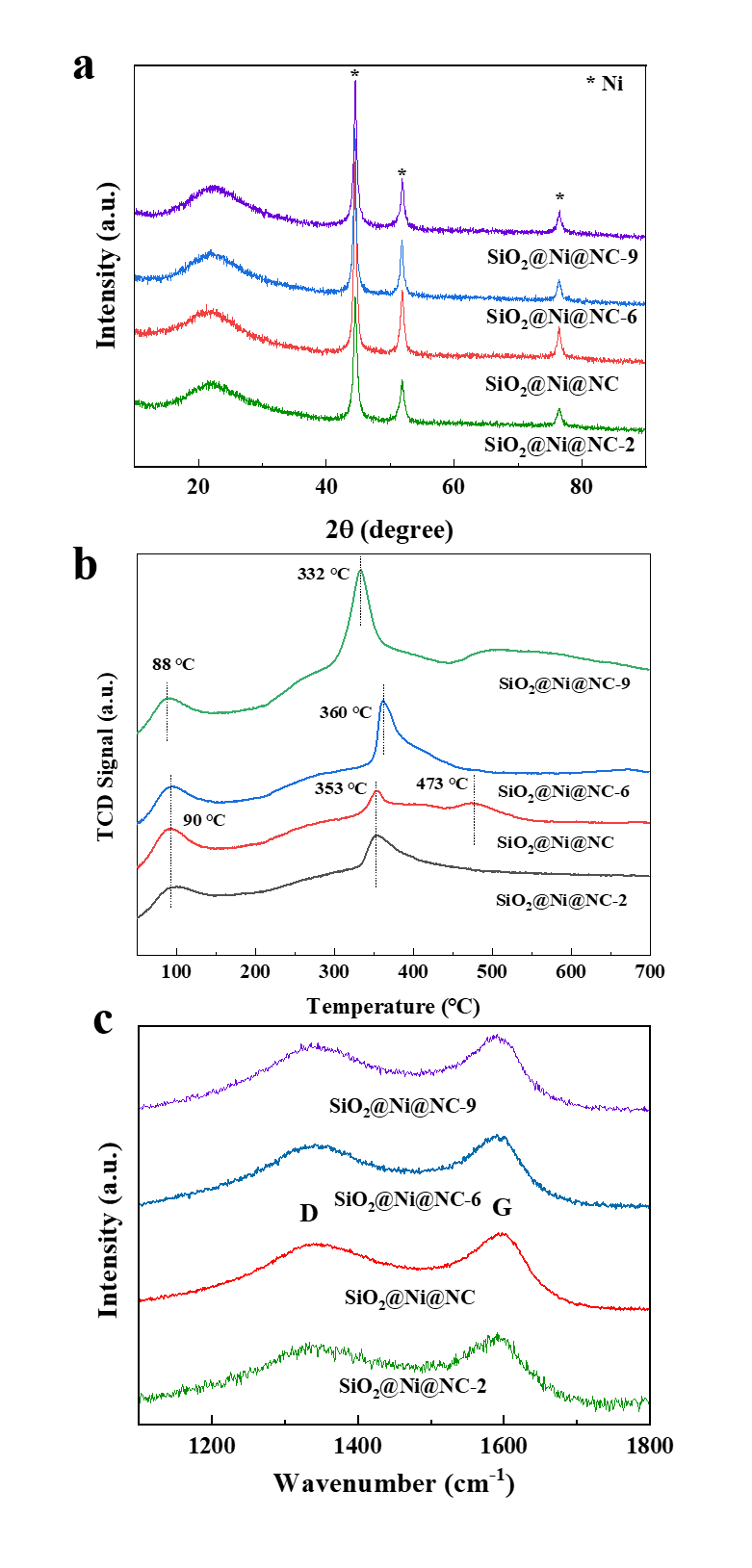


**Figure S17.** (a) XRD patterns, (b) H_2_-TPD and (c) Raman spectra of SiO_2_@Ni@NC with different NC layer thickness.


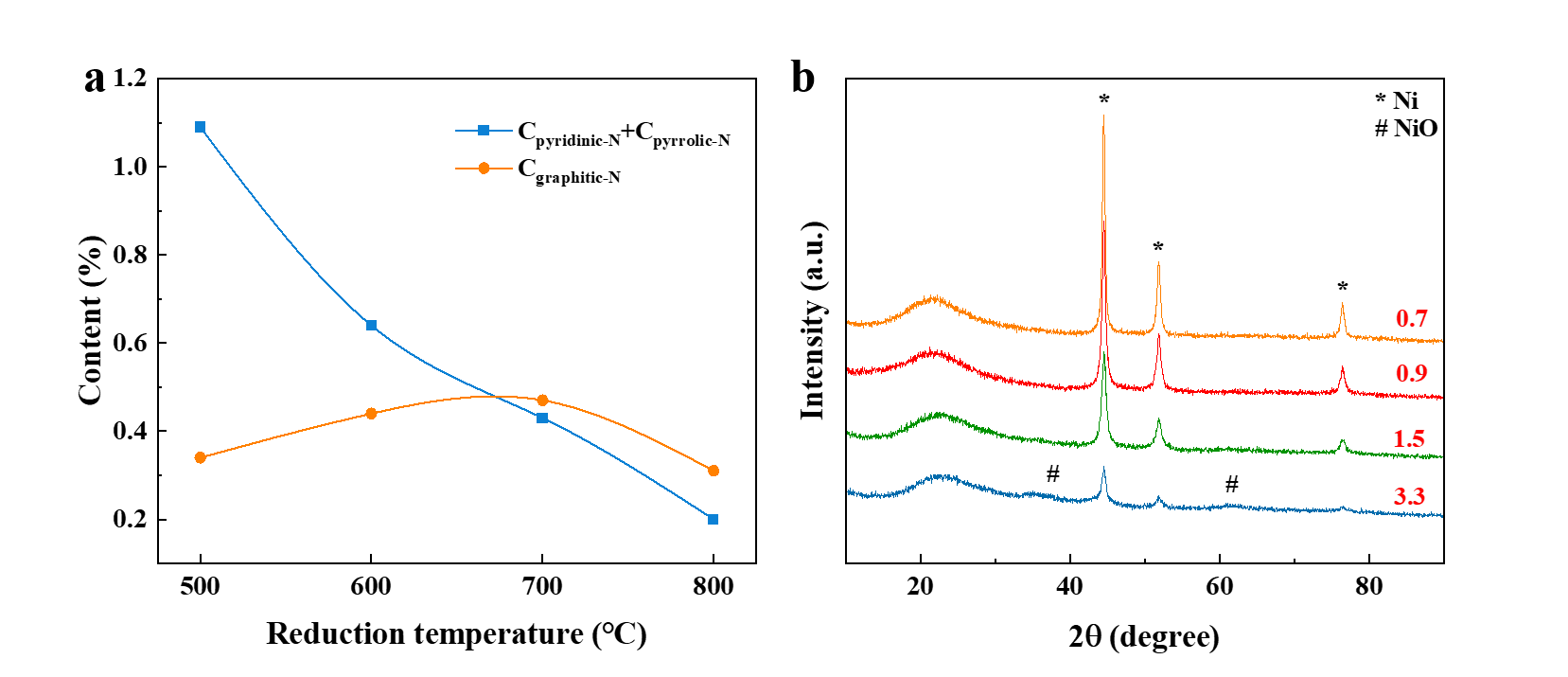


**Figure S18.** (a) Nitrogen content of SiO_2_@Ni@NC at different reduction temperatures; (b) XRD patterns of SiO_2_@Ni@NC with different (C_pyridinic-N_ + C_pyrrolic-N_):C_graphitic-N_ ratio.


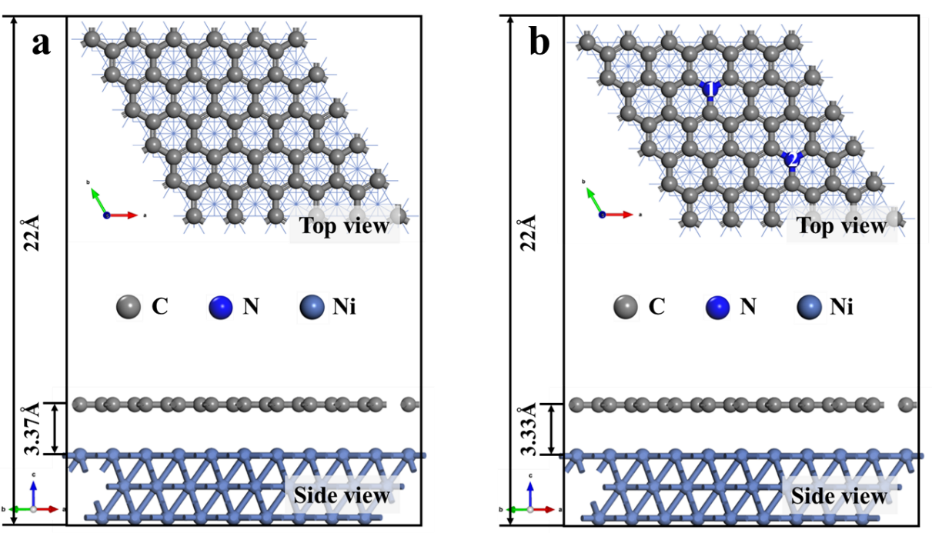


**Figure S19.** The side and top view of schematic models of (a) SiO_2_@Ni@C and (b) SiO_2_@Ni@NC.


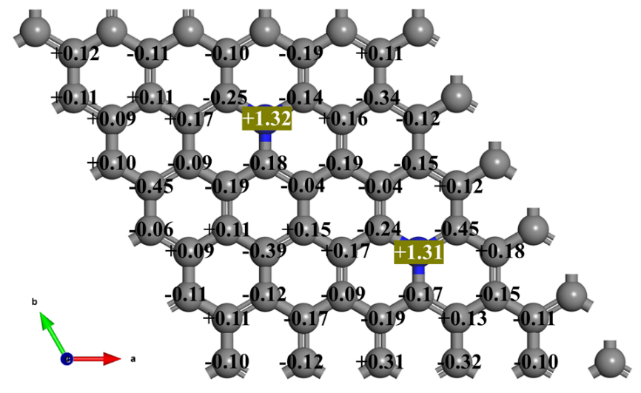


**Figure S20.** The Bader charge numbers of C and N atoms in doped carbon layer.


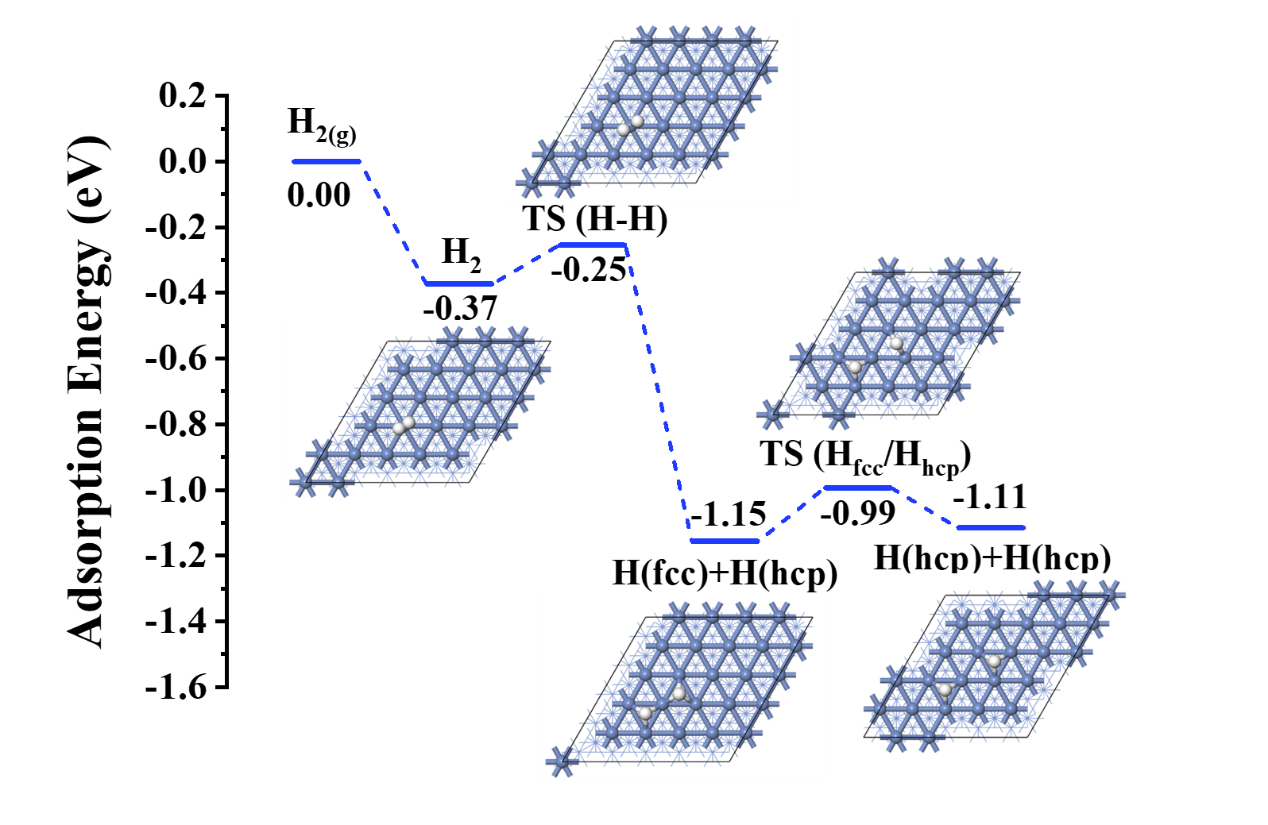


**Figure S21.** H_2_ dissociative adsorption and H diffusion over Ni (111).


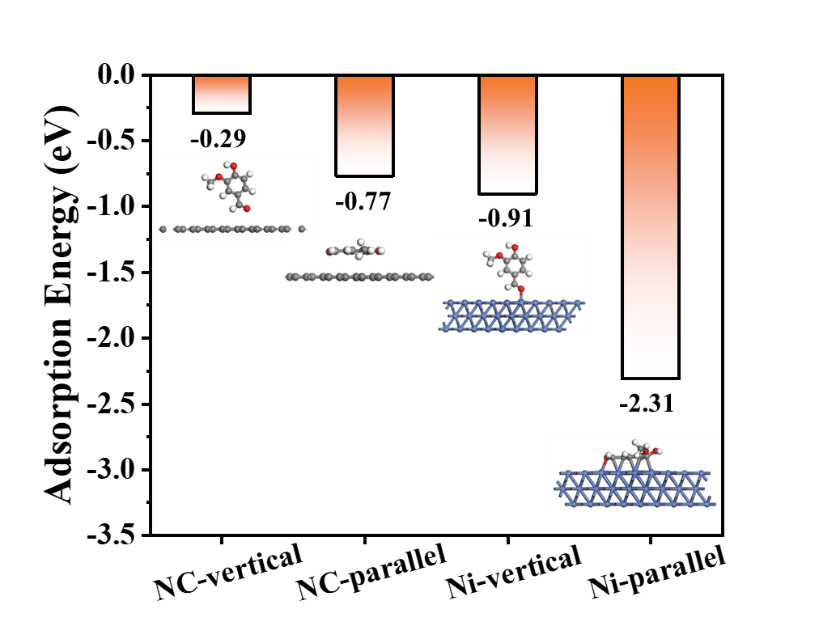


**Figure S22.** Adsorption configurations and energies of vanillin over NC and Ni (111).


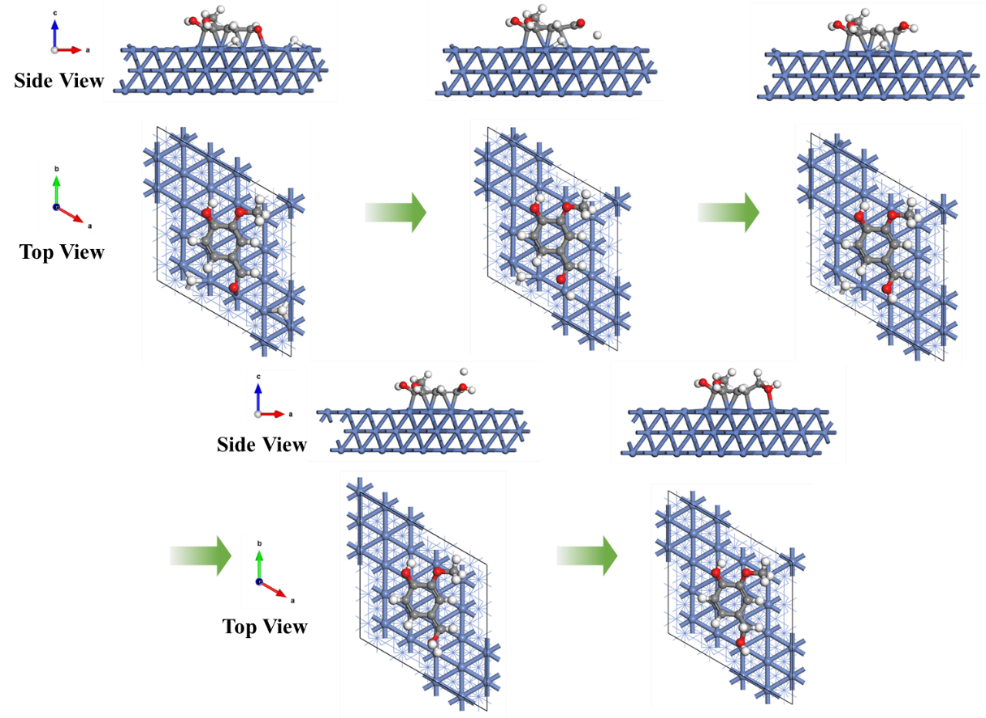


**Figure S23**. Side (above) and tope (below) views of all the optimized geometries for the reaction route R1.


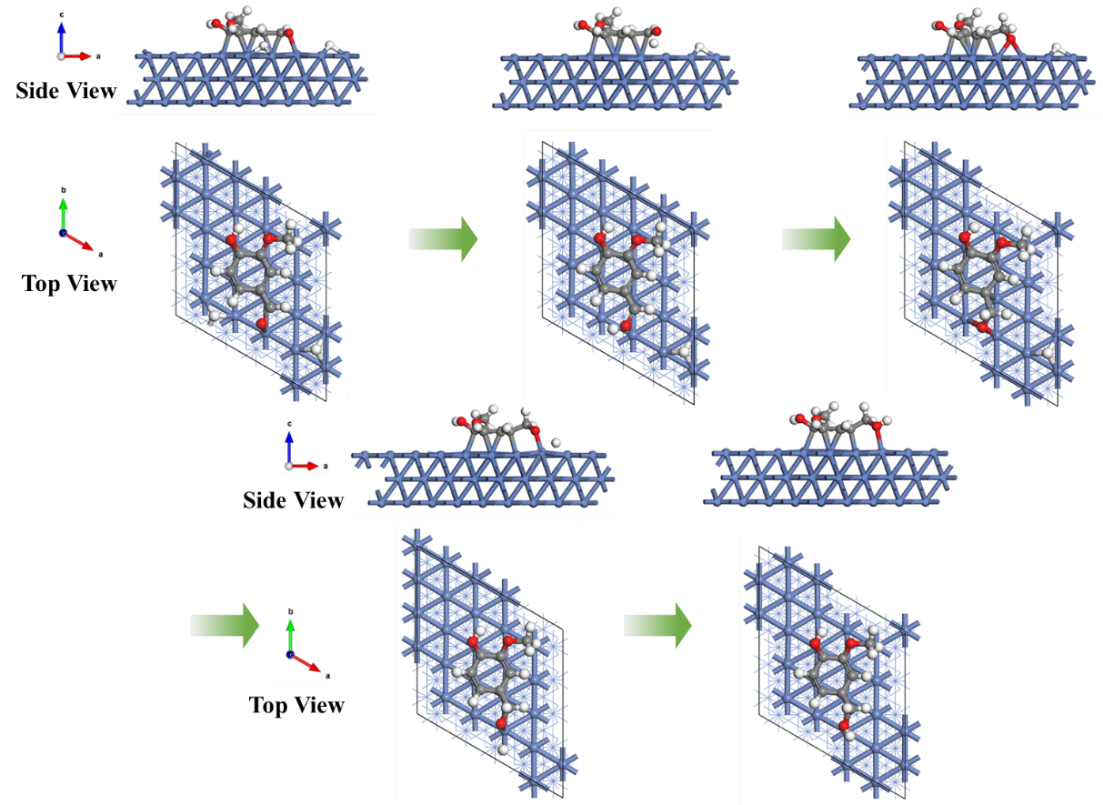


**Figure S24.** Side (above) and tope (below) views of all the optimized geometries for the reaction route R2.

**Table S1.** The content of organic elements in different samples.

| Entry | Sample | Content (%) | | |
| --- | --- | --- | --- | --- |
|  |  | N | C | H |
| 1 | SiO_2_@Ni | 0.06 | 0 | 0.60 |
| 2 | SiO_2_@Ni@NC-2 | 0.16 | 1.08 | 0.15 |
| 3 | SiO_2_@Ni@NC | 0.20 | 1.99 | 0.12 |
| 4 | SiO_2_@Ni@NC-6 | 0.30 | 6.15 | 0.16 |
| 5 | SiO_2_@Ni@NC-9 | 0.61 | 17.19 | 0.21 |

determined by elemental analyzer.

| **Table S2.** Summary of results of conversion of vanillin over different Ni-based catalysts.  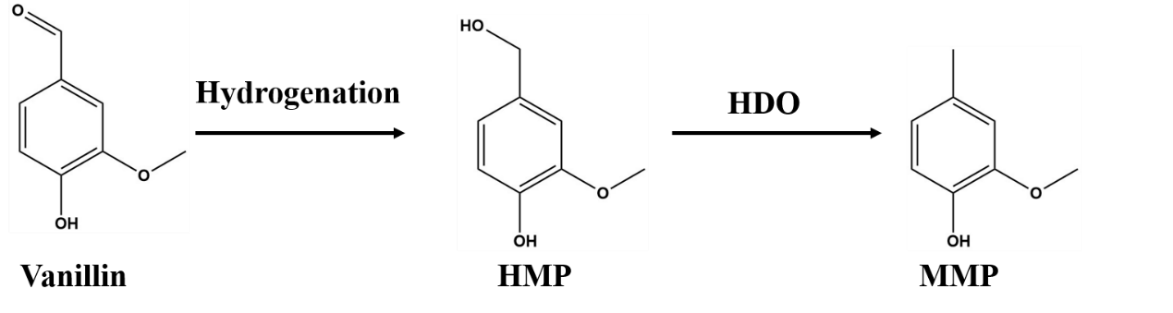 | | | | |
| --- | --- | --- | --- | --- |
| Entry | Catalyst | Reducing atmosphere | Conversion (%) | Selectivity of HMP (%) |
| 1 | SiO_2_@Ni(OH)_2_ | ‒ | 0 | 0 |
| 2 | SiO_2_@Ni(OH)_2_@RF | ‒ | 0 | 0 |
| 3 | SiO_2_@Ni | H_2_/Ar | 78.8 | 100 |
| 4 | SiO_2_@Ni@NC-H_2_/Ar | H_2_/Ar | 64.6 | 100 |
| 5 | SiO_2_@Ni@NC | N_2_ | 99.8 | 100 |
| 6 | SiO_2_@Ni@NC-Ar | Ar | 52.1 | 100 |
| Reaction conditions: vanillin (1 mmol), catalyst (30 mg), water (10 mL), H_2_ pressure (3.5 MPa), temperature (25 ℃), reaction time (2 h). | | | | |

**Table S3.** Structural properties of different samples.

| Entry | Physicochemical properties | Sample | |
| --- | --- | --- | --- |
|  |  | SiO_2_@Ni | SiO_2_@Ni@NC |
| 1 | TOF (h^-1^) | 8.3 | 20.7 |
| 2 | Ni dispersion (%) | 21.2 | 20.1 |
| 3 | Ni surface area (m^2^ g^-1^ sample) | 282.8 | 279.1 |
| 4 | Ni surface area (m^2^ g^-1^ Ni) | 141.4 | 133.1 |
| 5 | Active particle diameter (nm) | 4.8 | 5.1 |
| 6 | Cubic crystallite size (nm) | 3.9 | 4.1 |

determined by CO Chemisorption.

**Table S4.** ICP results of different samples.

| Entry | Sample | ICP-AES result (wt %) |
| --- | --- | --- |
| 1 | SiO_2_@Ni@NC | 21.0 |
| 2 | SiO_2_@Ni | 23.9 |
| 3 | used SiO_2_@Ni@NC after 5 cycles | 19.1 |
| 4 | used SiO_2_@Ni after 5 cycles | 5.4 |
| 5 | SiO_2_@Ni@NC-9 | 16.7 |
| 6 | SiO_2_@Ni@NC-6 | 19.9 |
| 7 | SiO_2_@Ni@NC-2 | 22.5 |

determined by ICP-AES.

**Table S5**. Chemical state of catalyst with different samples.

| Entry | Sample | Atomic (%) | | |
| --- | --- | --- | --- | --- |
|  |  | Ni 2p | C 1s | N 1s |
| 1 | SiO_2_@Ni@NC-2^a)^ | 6.1 | 10.9 | 0.2 |
| 2 | SiO_2_@Ni@NC^a)^ | 1.7 | 54.0 | 0.9 |
| 3 | SiO_2_@Ni@NC-Ar^b)^ | 2.3 | 33.9 | 0.8 |
| 4 | SiO_2_@Ni@NC-6^a)^ | 1.1 | 65.7 | 0.6 |
| 5 | SiO_2_@Ni@NC-9^a)^ | 1.7 | 69.8 | 0.4 |

determined by XPS.

^a)^ Treated under N_2_ atmosphere.

^b)^ Treated under Ar atmosphere.

**Table S6.** The adsorption energy of different slab models.

| Slab model | Adsorption energy (eV) | |
| --- | --- | --- |
|  | Vertical | Parallel |
| NC | -0.29 | -0.77 |
| Ni (111) | -0.91 | -2.31 |

**Table S7.** The energy barrier of different reaction routes.

| Reaction route | | | Energy barrier (eV) |
| --- | --- | --- | --- |
| R1 | Step 1 | V-CHOH | 1.16 |
|  | Step 2 | V-CH_2_OH | 2.1 |
| R2 | Step 1 | V-CH_2_O | 0.86 |
|  | Step 2 | V-CH_2_OH | 0.54 |

[1] G. Kresse, J. Furthmüller, *Phys Rev Lett.* **1996**, 54, 11169.

[2] J. P. Perdew, K. Burke, M. Ernzerhof, *Phys Rev Lett.* **1996**, 77, 3865.

[3] P. E. Blöchl, *Phys Rev Lett.* **1994**, 50, 17953.

[4] S. Grimme, J. Antony, S. Ehrlich, H. Krieg, *J Chem Phys.* **2010**, 132, 154104.

[5] V. Wang, N. Xu, J.-C. Liu, G. Tang, W.-T. Geng, *Comput Phys Commun.* **2021**, 267, 108033.

[6] G. Henkelman, B. P. Uberuaga, H. Jónsson, *J Chem Phys.* **2000**, 113, 9901.

[7] G. Henkelman, H. Jónsson, *J Chem Phys.* **2000**, 113, 9978.

[8] G. Henkelman, A. Arnaldsson, H. Jónsson, *Comp Mater Sci.* **2006**, 36, 354.
